# Processing changes when listening to foreign-accented speech

**DOI:** 10.3389/fnhum.2015.00167

**Published:** 2015-03-25

**Authors:** Carlos Romero-Rivas, Clara D. Martin, Albert Costa

**Affiliations:** ^1^Speech Production and Bilingualism, Center for Brain and Cognition, Universitat Pompeu FabraBarcelona, Spain; ^2^BCBL – Basque Center on Cognition, Brain and LanguageSan Sebastian, Spain; ^3^IKERBASQUE, Basque Foundation for ScienceBilbao, Spain; ^4^Institució Catalana de Recerca i Estudis AvançatsBarcelona, Spain

**Keywords:** ERPs, foreign-accented speech, adaptation, perceptual learning, lexical-semantic processing, P200, N400, P600

## Abstract

This study investigates the mechanisms responsible for fast changes in processing foreign-accented speech. Event Related brain Potentials (ERPs) were obtained while native speakers of Spanish listened to native and foreign-accented speakers of Spanish. We observed a less positive P200 component for foreign-accented speech relative to native speech comprehension. This suggests that the extraction of spectral information and other important acoustic features was hampered during foreign-accented speech comprehension. However, the amplitude of the N400 component for foreign-accented speech comprehension decreased across the experiment, suggesting the use of a higher level, lexical mechanism. Furthermore, during native speech comprehension, semantic violations in the critical words elicited an N400 effect followed by a late positivity. During foreign-accented speech comprehension, semantic violations only elicited an N400 effect. Overall, our results suggest that, despite a lack of improvement in phonetic discrimination, native listeners experience changes at lexical-semantic levels of processing after brief exposure to foreign-accented speech. Moreover, these results suggest that lexical access, semantic integration and linguistic re-analysis processes are permeable to external factors, such as the accent of the speaker.

## Introduction

Most of the studies addressing the processes behind spoken sentence comprehension have been conducted in the context of native speech. Although this is a reasonable strategy, conversations involving at least one foreign-accented speaker are becoming frequent due to increasing interest in foreign language learning and global mobility. In this context, two questions are fundamental. First, what are the acoustic/perceptual (or “bottom up”) processing challenges that listeners face when presented with foreign-accented speech? Second, to what extent are higher-level, lexical-semantic (or “top-down”) processes altered for these same types of foreign-accented stimuli? The present study aims at addressing some aspects of these two issues.

Early stages of speech comprehension, in which the incoming signal is acoustically mapped onto the listener's phonological repertoire, seem to be somewhat compromised when processing foreign-accented speech (Lane, 1963; Munro and Derwing, 1995a,b; Schmid and Yeni-Komshian, 1999; van Wijngaarden, 2001). This is because the phonological properties of foreign-accented speech often depart from those of the native listener. For instance, when a target phoneme in the second language (L2) does not exist in the speaker's native language, or when it is very similar to a native phoneme, foreign-accented speakers frequently substitute the L2 sound with a native sound (Wester et al., 2007). Moreover, variation in non-native speech is not restricted to segmental information, but it is also perceptible at the suprasegmental level; that is, variation is not only present at phonological levels, but also in the speaker's pitch and intonation contour (Gut, 2012). This is important, since word and sentence stress, as well as prosody and intonational deviations, are as important to intelligibility as segmental aspects (Fraser, 2000; Jilka, 2000). In addition, non-native speakers tend to be more variable than native speakers in their pronunciation (Nissen et al., 2007; Wade et al., 2007), meaning that sometimes they succeed in producing canonical sounds and sometimes they do not (Hanulíková and Weber, 2012). Such difficulty in language production could compromise conversational partners' semantic and syntactic processes (Goslin et al., 2012; Hanulíková et al., 2012). Finally, listeners use foreign accents as cues to categorize non-native speakers, modifying their mental representation about the speaker [e.g., modifying their expectations about the grammatical well-formedness of foreign-accented speech (Hanulíková et al., 2012); and relaxing their vowel categories more readily for foreign-accented speakers than for native speakers (Hay et al., 2006)].

In this article we address two main issues related to speech comprehension in foreign-accented contexts. First, we explore whether fast adaptations at the phonetic/acoustic and/or lexical level occur during speech comprehension. In particular, we ask whether a relatively short exposure to correct sentences (later in the experiment there will be semantic violations as well) pronounced with a foreign accent is sufficient to significantly improve the comprehension of the words in such utterances. As discussed below, we take the modulation of the P200 and N400 ERP components across the experiment as an index of improvement at phonetic/acoustic discrimination and word comprehension, respectively.

Second, we explore whether semantic processing is affected after listeners have gotten better at comprehending foreign-accented speech. We address whether the N400 (also associated to difficult semantic integration during semantic violations processing) and the P600 components (associated with the re-mapping of unexpected semantic features) are modulated by foreign-accented speech when semantic violations are present. This will inform us about whether after adapting to foreign-accented speech, semantic integration and meaning construction processes are compromised during the comprehension of foreign-accented speakers.

### Perceptual learning of foreign-accented speech

Despite the pervasive effects of foreign-accented speech on intelligibility (misidentification of words: Lane, 1963; Munro and Derwing, 1995a,b; van Wijngaarden, 2001) and comprehension^1^ (detecting mispronunciations and during sentence verification tasks: Munro and Derwing, 1995b; Schmid and Yeni-Komshian, 1999), native speakers improve their understanding of foreign-accented speech after brief exposure. After training with accented speech, native speakers are more accurate with the accent they were trained with in subsequent word transcription tests (Clarke, 2000; Weil, 2001; Bradlow and Bent, 2003). Clarke and Garrett (2004) presented English native speakers with English sentences uttered either by a native speaker of English or by a Spanish foreign-accented speaker of English, in the context of a probe word matching task. Clarke and Garrett (2004) observed that listeners were initially slower to respond to the Spanish-accented speech than to the native speech, but this difficulty decreased after 1 min of exposure. More recent studies have shown that this effect appears regardless of the speaker's baseline intelligibility, although the amount of exposure needed to achieve significant improvements varies depending on the strength of the accent, as well as on the listener's experience with a particular accent (Bradlow and Bent, 2008; Witteman et al., 2013). Moreover, adaptation is even present when foreign-accented speakers are inconsistent in their pronunciations (Witteman et al., 2014), but not when the accented variant forms are arbitrary instead of genuine (Weber et al., 2014). Importantly, accent learning occurs both with sentence- and word-length utterances, which suggests that listeners are sensitive to the global properties associated with accent, such as prosody and intonation contours, and also to segmental properties of speech that vary with accent (Sidaras et al., 2009).

A potential mechanism behind this adaptation is perceptual learning, a process which helps listeners to categorize ambiguous phonemes using lexical information. In an influential study, Norris et al. (2003) presented listeners with an ambiguous phoneme [?], midway between [f] and [s]. Listeners were exposed to the ambiguous phoneme in one of three training conditions (first: [?] version of 20 [f]-final words and the natural version of 20 [s]-final words; second: [?] version of 20 [s]-final words and the natural version of 20 [f]-final words; third: [?] was presented as the last phoneme in experimental non-words). After training, subjects were asked to categorize a range of ambiguous fricatives on a five step [εf]–[εs] continuum. Results showed that the categorization of these sounds shifted as a function of the training condition. That is, there were more [f] responses after exposure to ambiguous [f]-final and natural [s]-final words than after exposure to ambiguous [s]-final and natural [f]-final words (and vice versa). Most importantly, perceptual learning seemed to be absent when training with the ambiguous phoneme happened in a non-words context (see also: Davis et al., 2005). Norris et al. (2003) concluded that their results “do not show an increase in the listeners' ability to make phonetic discriminations. Instead, the results show that there was a change in the way an ambiguous phoneme was categorized, with the direction of that change determined by information that was only available from the lexicon” (p. 229). In addition, these changes generalize to words that have not been presented during the training phase (Davis et al., 2005; McQueen et al., 2006; Sjerps and McQueen, 2010). Thus, listeners would learn that the ambiguous phoneme is a representative form of the original phoneme, and this processing would be driven by lexical information.

Therefore, the first purpose of our study is to explore the temporal dynamics of the perceptual learning of foreign-accented speech (that is, the interaction between bottom-up, acoustic/phonetic processing challenges and top-down, lexical-semantic processes). Following Norris et al.'s (2003) conclusions, if perceptual learning does not entail an increase in the listeners' ability to make phonetic discriminations, then adaptation to a foreign accent should not appear during phonetic/acoustic extraction processes, but during lexical processing. For this purpose, we will explore the P200 and N400 ERP components modulation after exposure to foreign-accented speech (see below). Interestingly, Norris et al.'s (2003) results have been replicated recently using natural foreign phonemes (instead of an ambiguous phoneme artificially created in a laboratory; Sjerps and McQueen, 2010), and also in a foreign-accented context (Reinisch and Holt, 2014). This would suggest that adaptation to foreign acoustical properties might be guided, at least partially, by perceptual learning.

### The P200 component as an index of phonetic/acoustic processing

The P200 component (a positive deflection in the ERP wave peaking at around 200 ms after target presentation) has been often associated with the extraction of important acoustic features used for phonological and phonetic processing (Reinke et al., 2003; De Diego Balaguer et al., 2007). Interestingly, the amplitude of this component is positively correlated with the relative ease of this extraction process. For example, normal speech elicits a more positive P200 than degraded speech (Strauß et al., 2013). Hence, to the extent that extracting phonetic information from foreign-accented speech is more difficult than from native speech, one would expect the amplitude of the P200 to be smaller in a foreign-accented speech context.

In this study, we will explore whether exposure to foreign-accented speech across two experimental blocks has an effect on the extraction of the acoustic, pre-lexical features used for phonological processing. Indeed, this strategy has recently been followed by Rossi et al. (2013) in a different but related context. Rossi et al. (2013) conducted a study in which native speakers of German repetitively listened to Slovakian phonotactic regularities at the onset of pseudo-words. The results showed an increase in the amplitude of the P200 component after 3 days. However, Rossi et al. (2013) did not find differences in the amplitude of the P200 component between the pre-tests and post-tests in any of the 3 days of exposure. Since the P200 component is associated to the detection of relevant auditory cues (Reinke et al., 2003; De Diego Balaguer et al., 2007), Rossi et al.'s (2013) results suggest that listeners do not improve at detecting pre-lexical foreign features after brief exposure, but only after repetitive exposure to these pre-lexical regularities day after day. Therefore, if Rossi et al.'s (2013) observations on the learning of foreign phonotactic rules can be extended to the learning of foreign-accented speech phonetic variations, then we expect that the P200 component would not be modulated after brief exposure to foreign-accented speech. That would be suggesting that listeners do not improve at detecting relevant auditory cues for phonetic processing after brief exposure to foreign-accented speech.

### The N400 and P600 components as indices of lexical and semantic processing

The N400 component is a negative deflection in the ERP wave peaking at around 400 ms, and that usually shows a centro-posterior scalp distribution. The N400 component is sensitive to a range of features such as: (a) sublexical variables of words, like orthographic similarity to other words in the language [words with more orthographic neighbors elicit larger N400s (Holcomb et al., 2002; Laszlo and Federmeier, 2011)]; (b) lexical variables, such as word frequency, or concrete vs. abstract concepts (Kroll and Merves, 1986; Smith and Halgren, 1987; Van Petten and Kutas, 1991; West and Holcomb, 2000; Gullick et al., 2013); (c) semantic relationships among words (Neely, 1991; McNamara, 2005; Van Petten and Luka, 2006); and (d) cloze probability during sentence comprehension (Kutas and Hillyard, 1984; Kutas et al., 1984; DeLong et al., 2005, 2012; Thornhill and Van Petten, 2012; Wlotko and Federmeier, 2013).

In the present study—following Norris et al.'s (2003) and Davis et al.'s (2005) conclusions—we expect that a top-down mechanism will allow listeners to retune sublexical features during foreign-accented speech. As this mechanism is supposed to be driven by lexical information, we expect to capture a reduction in the N400 component for foreign-accented speech across experimental blocks.

Regarding semantic processing, there is scarce evidence and a lack of agreement on the effects of foreign-accented speech on the N400 component. Hanulíková et al. (2012) presented listeners with sentences uttered either by a native speaker of Dutch or by a Turkish foreign-accented speaker of Dutch. They found a similar N400 effect for semantic violations (e.g., “It was very cold last night, so I put a thick *blanket/evening* on my bed”) during both native and foreign-accented speech, although it was more widely distributed over the scalp during foreign-accented speech comprehension. Hanulíková et al. (2012) concluded that native listeners had no problem understanding foreign-accented speech, as indicated by almost equivalent electrophysiological responses to semantic violations produced by native and foreign-accented speakers. On the other hand, Goslin et al. (2012) presented listeners with correct sentences uttered by native, regional (a different dialect), and foreign speakers of English. Final words uttered by foreign-accented speakers elicited reduced N400 components when compared to both native and regional accented conditions. Goslin et al. (2012) concluded that because of the degraded signal (due to foreign accent), native listeners hearing foreign speakers would rely on top-down processes (i.e., paying more attention and placing more effort on anticipating upcoming words) in order to understand the incoming speech. That is, Hanulíková et al. (2012) and Goslin et al. (2012) reached two different conclusions. While Hanulíková et al. (2012), proposed that global meaning was not affected by foreign-accented speech, Goslin et al. (2012) suggested that listeners had to use top-down processes in order to compensate for a comprehension deficit during foreign-accented speech.

The second purpose of this study is to clarify this issue, by including semantic violations in the second part of the experiment. More concretely, we will explore whether exposure to foreign-accented speech has an effect on further linguistic processes, such as semantic integration (as indexed by the N400) and meaning re-analysis (as indexed by the P600).

The P600 component is a positive-going deflection in the ERP wave which peaks at a later time point than the N400, lasting until approximately 900 ms after word onset. The P600 is considered an index of a second stage of processing, involving a continued analysis of the current word with respect to its context and to the information stored within long-term memory (Kuperberg et al., 2011). For instance, a P600 effect is observed for words that are highly semantically implausible with respect to their context (Kuperberg, 2007; Van de Meerendonk et al., 2010), or by words that require deeper causal inferences (Burkhardt, 2006, 2007).

The present knowledge regarding the modulation of the P600 component in foreign-accented contexts is limited to Hanulíková et al.'s (2012) study. Interestingly, in their study, the P600 component was sensitive to gender agreement errors only when sentences were presented in a native accent, but not when they were presented with a foreign accent. Hanulíková et al. (2012) concluded that listeners had “learned” to be tolerant to these grammatical mistakes when presented in foreign-accented speech^2^.

Summarizing, the present study aims to explore two main questions. First, what are the specific adaptations that native speakers perform to deal with foreign-accented speech? More concretely, we explored whether native speakers experience a change in the acoustic/phonetic processing after brief exposure to foreign-accented speech or, on the other hand, whether the usual improvement in comprehension observed during exposure to foreign-accented speech is dependent on top-down, lexical-semantic processes. The second question is whether, after these adaptations are acquired, further linguistic processes are affected by foreign-accented speech—such as semantic integration and meaning re-analysis. To address these issues, Spanish native speakers were presented with a large set of sentences either produced with a native accent or with a foreign one. In the first block of the experiment we used standard (correct) sentences (meaningful and unsurprising sentences). In the second block, standard sentences were randomly mixed with sentences containing a semantic violation. The EEG was recorded during the experiment and time-locked ERPs were explored. We focused our analysis on the P200, N400, and P600 components elicited by the first, critical and final word of each sentence (see Table 1 for examples).

**Table 1:** **Examples of sentences with English translation**.

Mi desayuno favorito es tostadas con mermelada y un *café/hospital* con mucha leche.
“My favorite breakfast is a toast with marmalade and a *coffee/hospital* with a lot of milk.”
Cuando mi sobrina duerme en mi piso siempre le leo un *libro/pan* por la noche.
“When my niece sleeps in my flat I always read to her a *book/bread* by the night.”
María tuvo que imitar a un *pirata/comercio* en la fiesta.
“María had to imitate a *pirate/store* in the party.”
Para ir a Barcelona siempre pasamos por un *túnel/piano* en la autovía.
“Coming to Barcelona we always cross a *tunnel/piano* in the highway.”

*ERPs were obtained during the first, critical and final word of the sentences (underlined words). Critical words are in italics. Semantic violations were only introduced during Block 2*.

Following Norris et al.'s (2003) conclusions, if perceptual learning does not entail an increase in the listener's ability to make phonetic discriminations, we expect a lower P200 amplitude for foreign-accented as compared to native speech across the whole experiment. Moreover, if listeners retune sublexical and/or supralexical features of speech using a top-down mechanism driven by lexical information (Norris et al., 2003; Davis et al., 2005), we expect that the N400 amplitude for foreign-accented speech will decrease across experimental blocks. Also, if linguistic processes such as semantic processing and meaning re-analysis are affected by foreign-accented speech even after exposure to the accented speakers, we expect that the N400 and P600 effects for semantic violations in the second block will be modulated by the speaker's accent. Based on previous literature (Hanulíková et al., 2012), we expect an N400 effect for semantic violations distributed more widely over the scalp during foreign-accented speech comprehension, as compared to native speech comprehension. In addition, Hanulíková et al. (2012) observed a P600 effect for grammatical mistakes during native speech comprehension, an effect that was missing during foreign-accented speech comprehension. Thus, we also expect a modulation of the P600 effect depending on the speaker's accent.

## Materials and methods

### Participants

Twenty native speakers of Spanish (12 women, all right handed, mean age = 24.1 years, range = 19–35 years) participated in this experiment in return for monetary compensation (10€/h). Participants were mostly from Catalonia (hence, from the same dialectal variation), and Spanish was their dominant language (they would speak Spanish to their parents, and they would use Spanish >70% of the time when interacting with other people). None of them reported any hearing or neurological impairments. Before the beginning of the experiment, subjects gave their written informed consent.

### Stimuli

The experimental stimuli consisted of a set of 208 sentences uttered by four native and four foreign-accented speakers of Spanish. Each sentence was recorded four times: a standard version spoken by one of the native speakers, a standard version spoken by one of the non-native speakers, a version containing a semantic violation in the critical word spoken by one of the native speakers, and a version containing a semantic violation in the critical word spoken by one of the foreign accented speakers (resulting in 832 sentences; for auditory samples of some experimental sentences, see Supplementary Material). Each quartet (the four versions of each sentence) was recorded by a native and a foreign-accented speaker. Critical words were always nouns in a mid-sentence position (range = 1–5 words between critical word and final word, mean = 2.34 words; *SD* = 0.9), balanced for phonological length [mean for the Standard condition = 6.66 phonemes (*SD* = 1.91); mean for the Semantic Violation condition = 6.48 phonemes (*SD* = 2.11); *p* = 0.37] and frequency [mean for the Standard condition = 3.08 (*SD* = 0.58); mean for the Semantic Violation condition = 3.09 (*SD* = 0.58); *p* = 0.35]. Logarithmic values for word frequency were extracted from the SUBTLEX-ESP corpus (Cuetos et al., 2011). In addition, the critical words of each sentence in Standard condition and Semantic Violation condition always started with a different phoneme. The first and final words of each sentence were also analyzed (see Table 1).

The native languages of the foreign speakers were French, Greek, Italian, and Japanese. The decision to use these speakers was rooted in the aim to test the main effect of foreign accented speech, independently of the native language of the foreign speakers and the similarities between Spanish and those other languages. We looked for variability in the speakers' accent on purpose, so any effect on comprehension would be due to foreign-accented speech, and not to a specific error pronounced with a specific accent (for similar methodological choices, see Schmid and Yeni-Komshian, 1999; Floccia et al., 2009)^3^. Nevertheless, we controlled for stress patterns in the pronunciation of foreign-accented speakers, in order to avoid effects such as those of weird stress shifts or irregular metrics on the ERP components (Rothermich et al., 2012). In order to do so, foreign-accented speakers were presented with native-accented versions of the sentences before their recordings. In any case, Reinisch and Weber (2012) showed that native listeners can adapt to stress errors produced by foreign-accented speakers after brief exposure.

**Figure 1:**
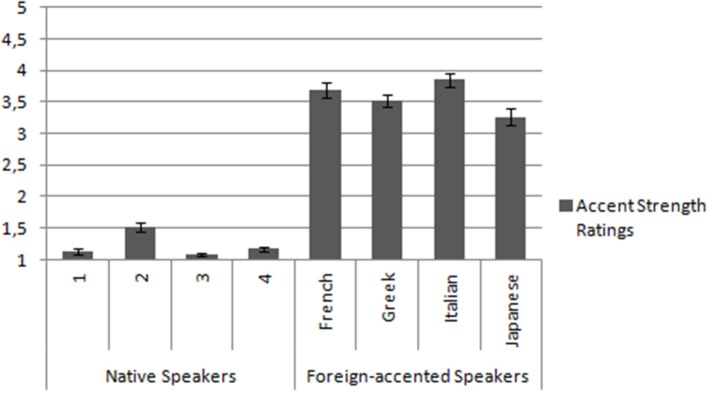
**Accent strength ratings**. Ratings were from 1 (native speech) to 5 (the speaker has a very strong foreign accent).

Accent strength and intelligibility of the eight speakers were tested by an independent sample of 27 native speakers of Spanish (19 women, mean age = 22.93 years, range = 18–38 years). Participants in the pre-tests were also mostly from Catalonia, and Spanish was their dominant language as well (they would speak Spanish to their parents, and they would use Spanish >70% of the time when interacting with other people). These pre-tests were run in order to ensure that native and foreign-accented speakers were perceived differently, and that, beyond this difference, they were all understandable. Participants carried out two tasks. During the first task, they had to listen to the experimental sentences and rate them from 1 (native speech) to 5 (the speaker has a very strong foreign accent). For the second task, subjects had to write down the final word of each sentence (comprehension task). Regarding the first task, we carried out a repeated measures ANOVA including the within subject factors Accent (native, foreign) and Speaker (each of the eight speakers). A significant effect of Accent was obtained [*F*_(1, 26)_ = 793.93, *p* < 0.001], revealing that foreign speakers' accents (mean = 3.58, *SD* = 0.2) were evaluated as stronger than native speakers' accent (mean = 1.22, *SD* = 0.07). We also obtained a significant effect of Speaker [*F*_(3, 24)_ = 7.03, *p* < 0.01], and a significant interaction between the two factors [*F*_(3, 24)_ = 30.82, *p* < 0.01]. Planned comparisons revealed that each native speaker was rated significantly less accented than each non-native speaker. Also, between native speakers only speaker number 2 was rated as significantly more accented than the rest. Among foreign accented speakers only the Japanese one was rated as significantly less accented than the rest (for further details, see Figure 1). Regarding the second task, participants recognized the last word of the sentences 100 per cent of the times both for the native and for the foreign accented speakers, and did not report any difficulties in understanding the sentences. Based on these results, we can conclude that native and foreign-accented speakers were perceived differently, although all of them were understood^4^.

Sentences were recorded and edited with Audacity (© Audacity Team), at 44.1 kHz, 32 bits and in stereo sound. Each speaker received a list containing the experimental sentences in both versions (standard and containing semantic violations), randomized in order to avoid undesirable effects, such as different speech rates and voice intensities for first and second presentations of the same sentence context. As mentioned before, foreign-accented speakers were presented with native-accented versions of the sentences before their recordings, in order to minimize possible differences in speech rate and prosody. Nevertheless, there were differences in the mean duration of the whole sentence (native speech = 3311.24 ms, *SD* = 542.34; foreign accented speech = 4149.21 ms, *SD* = 687.64; *p* < 0.001), mean duration of critical words (native speech = 358.36 ms, *SD* = 108.93; foreign accented speech = 450.14 ms, *SD* = 137.95; *p* < 0.001), and mean duration of final words (native speech = 474.21 ms, *SD* = 144.33; foreign accented speech = 521.93 ms, *SD* = 150.67; *p* < 0.001). Following Goslin et al. (2012), no attempt was made to control or adjust the temporal features of the stimuli, as longer productions are an inherent part of foreign-accented speech (see also Hanulíková et al. (2012) for differences in sentences and critical words durations across accents).

In addition, we analyzed the acoustic features of the critical words using Praat (Boersma and Weenink, 2001). More concretely, we measured intensity (dB), and *f*_0_ mean and range (Hz) of the critical words of the 208 experimental sentences (see Supplementary Material Figure 5, for spectrographic representations of some sentences). We expected differences between native and foreign-accented speech in *f*_0_ related values, since variation in the speaker's pitch and intonation contour is a common feature of foreign-accented speech (Gut, 2012). Repeated measures ANOVA were conducted for each measure, including the factors Accent (native, foreign) and Semantic Status (standard, semantic violation). In the intensity analysis we did not observe any significant effect or interaction. In the *f*_0_ mean analysis, we observed a main effect of Accent [*F*_(1, 207)_ = 6.50; *p* < 0.05], revealing a higher *f*_0_ mean for foreign-accented speech (213.95 Hz) than for native speech (207.27 Hz). We also observed a main effect of Semantic Status [*F*_(1, 207)_ = 4.15; *p* < 0.05], showing a higher *f*_0_ mean for semantic violations (212.01 Hz) than for standard critical words (209.21 Hz). Importantly, the interaction between the two factors was not significant [*F*_(1, 207)_ < 1; *p* = 0.91]. Finally, in the *f*_0_ range analysis we observed a main effect of Accent [*F*_(1, 207)_ = 64.87; *p* < 0.001], revealing a wider *f*_0_ range during foreign-accented speech (96.03 Hz) than during native speech (77.13 Hz). These differences will be discussed later on (see Discussion).

Four experimental lists were created, each of them containing only one version of the 208 experimental sentences. There were two blocks in each list, although subjects were not warned about this characteristic. During the first block, subjects listened to only standard sentences (64 sentences, 32 spoken by the native speakers, 32 spoken by the non-native speakers, eight sentences per speaker, all sentences randomized). We chose to use only eight sentences per speaker because both improvement at foreign-accented speech comprehension (Clarke and Garrett, 2004) and perceptual learning (Norris et al., 2003) appear after very brief exposure to speech. During the second block, subjects listened to standard sentences and sentences containing semantic violations (144 sentences, 36 standard sentences spoken by native speakers, 36 standard sentences spoken by foreign-accented speakers, 36 sentences containing semantic violations spoken by native speakers, 36 sentences containing semantic violations spoken by foreign-accented speakers, with nine sentences per speaker and condition, all sentences randomized). The presentation of the standard sentences in the first or the second block was counterbalanced across subjects.

### Procedure

Participants were seated in front of a computer screen, in a soundproof room. They were asked to listen carefully in order to comprehend all sentences during a passive listening task. We did not provide any information about the speakers or their accents, only telling the participants that they will be listening to people speaking in an everyday context. The experiment was run on E-Prime 2.0. Sentences were presented binaurally via headphones at a constant, comfortable listening level set by the experimenters. Each trial started with a fixation point, presented 1000 ms before the onset of the auditory sentences and remained on the screen until 1000 ms after sentence offset. Participants were asked to stare at the fixation point and to avoid blinking throughout the auditory sentence presentation. Participants controlled initiation of the next trial by pressing the space bar. They were told to rest between trials if needed. The whole experiment lasted approximately 25 min.

### EEG recordings and analysis

The EEG signal was recorded from 64 active electrodes (impedance was kept below 10 kΩ) mounted in an elastic cap, at standard 10–20 locations. The on-line reference electrode was attached to the left mastoid, and re-referenced off-line to the mastoid average. Lateral eye movements were recorded with an electrode beside the right eye, and eye blinks were recorded with two electrodes, one above and the other below the right eye. EEG signal was filtered on-line with a 0.1–100 Hz bandpass filter and digitized at 500 Hz. EEG epochs were time-locked to the first word and the final word of each sentence (either coming from a correct sentence context or from a context containing a semantic violation), as well as to the critical words (those words manipulated to elicit a semantic violation in the experimental condition). Thereby we extracted the segments at 200 ms before and lasting until 1200 ms after the onset of each analyzed word. EEG waveforms were baseline corrected to a 200 ms pre-onset baseline, and averaged per participant and condition. Mean amplitudes in specific time windows were analyzed with repeated measures ANOVAs, analyzing three regions: frontal (F3, Fz, F4, FC5, FC3, FC1, FC2, FC4, and FC6), central (C3, C1, Cz, C2, C4, CP3, CP1, CP2, and CP4) and posterior (P5, P3, P1, Pz, P2, P4, P6, PO3, and PO4).

Statistical analyses focused on three main time windows. For the P200 effect we established an early time window (150–250 ms, based on previous literature; see, e.g., Rossi et al., 2013; Strauß et al., 2013). We analyzed the P200 component only on the first word of the sentences, since this component wanes in the ERP signal of words embedded in spoken sentences (e.g., see the comparison of spoken vs. written sentence final words in Kutas and Federmeier's (2001), Figure 1). A similar strategy has been used by Strauß et al. (2013): in a study on lexical expectations under degraded speech, Strauß et al. (2013) analyzed the P200 component only on the first word of spoken sentences. For the N400 effect we established an intermediate time window (250–600 ms, based on previous literature; see, e.g., Lau et al., 2008). The N400 component was analyzed on the first, critical and final words of each sentence. Finally, for the P600 component we established a late time window (600–900 ms, based on previous literature; see, e.g., Brouwer et al., 2012). The P600 component was analyzed for the critical word of each sentence, since it indexes semantic re-analysis processes, and we did not consider any hypothesis about this component for the first or final words of the sentences.

All effects and interactions including a variable with three factors were corrected for sphericity using the Greenhouse-Geisser correction. In addition, we used the Bonferroni correction for *post-hoc* analyses.

## Results

### P200: acoustic/phonetic processing

As argued in the introduction, modulations of the P200 component could be taken as an index of improvements in the extraction of acoustic/phonetic information during foreign-accented speech comprehension. To assess this issue we compared the amplitude of the P200 component for the first word of the sentences across the two experimental blocks. The repeated measures ANOVA for the P200 effect (150–250 ms) only included the first word of the sentences. The analysis included the factors Topography (frontal, central, posterior), Block (first, second) and Accent (native, foreign). We obtained a significant effect of Topography [*F*_(2, 38)_ = 9.11; *p* < 0.01], Accent [*F*_(1, 19)_ = 6.99, *p* < 0.05], and a significant interaction between the three factors [*F*_(2, 38)_ = 4.88, *p* < 0.05]. The *post-hoc* analysis for the interaction showed that the amplitude of the P200 component was similar for the two blocks in both native [*t*_(38)_ = 0.59, *p* = 1] and foreign-accented speech [*t*_(38)_ = 0.41, *p* = 1]. Furthermore, words produced with native speech elicited a more positive P200 amplitude than words produced with foreign-accented speech. This was the case for both block 1 [*t*_(38)_ = 4.60, *p* < 0.001] and block 2 [*t*_(38)_ = −3.82, *p* < 0.01]^5^. Native speech elicited a more positive mean amplitude than foreign-accented speech in the three topographic regions [frontal region: *t*_(38)_ = 3.15, *p* < 0.01; central region: *t*_(38)_ = 5.17, *p* < 0.001; posterior region: *t*_(38)_ = 3.41, *p* < 0.01].

**Figure 2:**
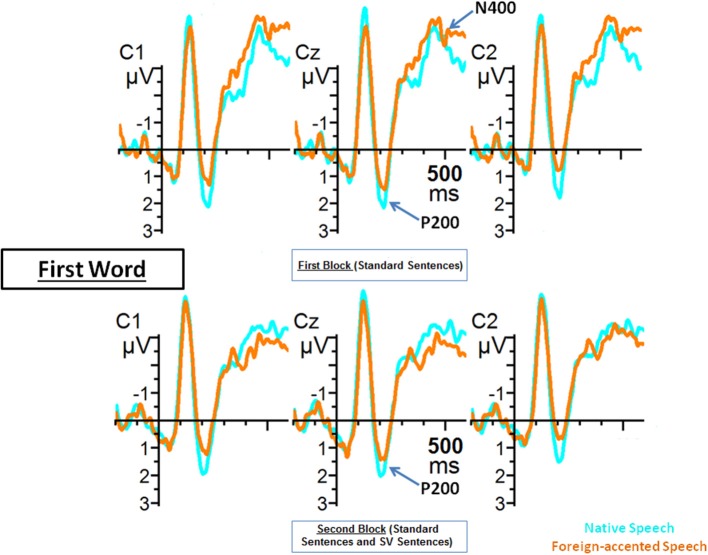
**Grand average ERPs from C1, Cz and C2 electrodes for the first word of the sentences of Blocks 1 and 2 during native (blue line) and foreign-accented (orange line) speech**. Grand average images were extracted at 200 ms before (baseline) and lasting until 600 ms after the onset of the words.

In sum, this analysis revealed that the mean amplitude of the P200 component for native speech was more positive than for foreign-accented speech across the experiment (Figure 2). This suggests that the extraction of phonetic/acoustic information was easier during native speech as compared to foreign-accented speech comprehension throughout the experimental session.

### N400: lexical-semantic processes

We carried out two repeated measures ANOVAs for the N400 component (250–600 ms). The first repeated measures ANOVA for the N400 component included the factors Topography (frontal, central, posterior), Position (first word, critical word, final word), Block (first, second) and Accent (native, foreign). Only correct sentences were included in this analysis. Our aim was to investigate whether listeners used specific lexical mechanisms in order to reach to a better comprehension of foreign-accented speech across the experimental session.

In this analysis we obtained significant effects for Topography [*F*_(2, 38)_ = 7.91, *p* < 0.01], Position [*F*_(2, 38)_ = 12.90, *p* < 0.001], and Accent [*F*_(1, 19)_ = 9.27, *p* < 0.01]. Significant interactions between Topography and Position [*F*_(4, 76)_ = 13.16, *p* < 0.001], and between Block and Accent [*F*_(1, 19)_ = 3.88, *p* < 0.05] were also obtained. Importantly, *post-hoc* analysis of the interaction between Block and Accent revealed differences between native and foreign-accented speech in the N400 mean amplitude only in the first block of the experiment [first block, *t*_(19)_ = 2.79, *p* < 0.05; second block, *t*_(19)_ = 0.29, *p* = 1]. Furthermore, while correct sentences in the native language elicited the same N400 amplitude across the experiment [*t*_(19)_ = 0.49, *p* = 1], foreign-accented sentences elicited a less negative N400 amplitude in the second block as compared to the first block [*t*_(19)_ = −2.85, *p* < 0.05].

*Post-hoc* analysis of the interaction between Topography and Position showed that in the frontal region there were no differences between word positions in terms of the N400 mean amplitude. In the central region there were significant differences between first and critical words [*t*_(76)_ = −4.59, *p* < 0.001], first and final word [*t*_(76)_ = −4.72, *p* < 0.001), and critical and final words [*t*_(76)_ = −3.29, *p* < 0.05]. In the posterior region there were also significant differences between first and critical words [*t*_(76)_ = −4.16, *p* < 0.001], first and final words [*t*_(76)_ = −4.22, *p* < 0.001], and critical and final words [*t*_(76)_ = −3.01, *p* < 0.05].

Although we obtained a significant interaction between Block and Accent, we wanted to carry out a deeper exploration of these results. More concretely, we examined whether the adaptation on the N400 component was present in the three word positions. Crucially, it was. During native speech comprehension, there were no significant differences between block 1 and 2 [first word: *t*_(19)_ = 0.13, *p* = 0.89; critical word: *t*_(19)_ = 0.47, *p* = 0.64; final word: *t*_(19)_ = 0.42, *p* = 0.68]. However, during foreign-accented speech comprehension, the mean amplitude of the N400 component was significantly reduced during block 2 as compared to block 1 [first word: *t*_(19)_ = 2.11, *p* < 0.05; critical word: *t*_(19)_ = 2.17, *p* < 0.05; final word: *t*_(19)_ = 2.21, *p* < 0.05]. In addition, while foreign-accented speech elicited a more negative N400 amplitude than native speech in block I [first word: *t*_(19)_ = 2.11, *p* < 0.05; critical word: *t*_(19)_ = 2.15, *p* < 0.05; final word: *t*_(19)_ = 2.21, *p* < 0.05], this difference disappeared in block 2 [first word: *t*_(19)_ = 0.50, *p* = 0.62; critical word: *t*_(19)_ = 0.19, *p* = 0.85; final word: *t*_(19)_ = 0.97, *p* =0.34].

**Figure 3:**
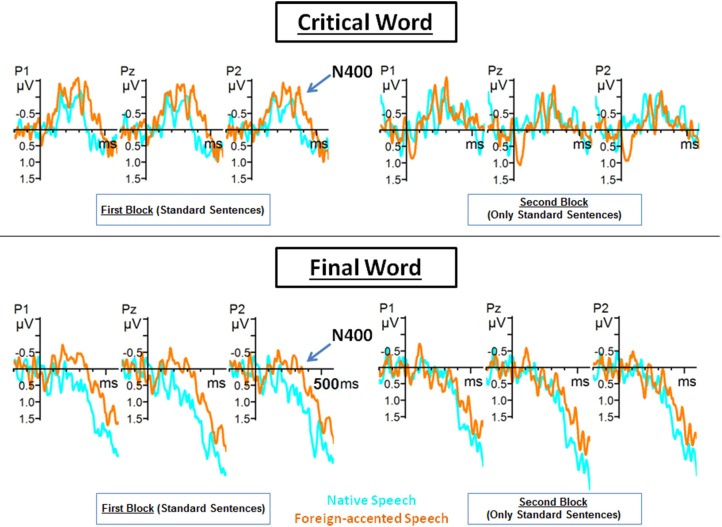
**Grand average ERPs from P1, Pz and P2 electrodes for the critical and final words of the correct sentences of Blocks 1 and 2 during native (blue line) and foreign-accented (orange line) speech**. Grand average images were extracted at 200 ms before (baseline) and lasting until 600 ms after the onset of the words.

In sum, this analysis revealed that during the first experimental block, foreign-accented speech elicited a more negative N400 mean amplitude than native speech comprehension did. However, during the second block, this difference disappeared—words uttered by native and foreign-accented speakers elicited similar N400 mean amplitudes (Figure 3). Importantly, this effect did not depend on word position. These results suggest that lexical-semantic processing of foreign-accented speech improved by the second experimental block.

In the second analysis performed on the N400 mean amplitudes, we included the sentences of the second experimental block, both correct sentences and sentences containing semantic violations (recall that no semantic violation was encountered during the first experimental block). Since the goal of this analysis was to explore the integration of semantic violations, we included only the critical words (those which could contain the semantic violation) of each sentence (and not the first and last words). The repeated measures ANOVA for the critical words of the second experimental block included the factors Topography (frontal, central, posterior), Accent (native, foreign), and Semantic status (standard, semantic violation). The motivation for this analysis was to explore whether foreign-accented speech affected semantic integration processes after the perceptual learning of the foreign accents. The analysis revealed a main effect of Semantic status [*F*_(1, 19)_ = 4.25, *p* < 0.05], and significant interactions between Topography and Semantic status [*F*_(2, 38)_ = 7.11, *p* < 0.05], Accent and Semantic status [*F*_(1, 19)_ = 5.41, *p* < 0.05], and between the three factors, Topography, Accent and Semantic status [*F*_(2, 38)_ = 3.69, *p* < 0.05]. Importantly, *post-hoc* analysis of the three-way interaction showed that in the frontal region, the difference between standard and semantic violation conditions was only significant for foreign-accented speech [*t*_(38)_ = 4.43, *p* < 0.001]. The same effect was observed in the central region [*t*_(38)_ = 4.49, *p* < 0.001]. In the posterior region, there were significant differences between the standard condition and semantic violations for both native [*t*_(38)_ = 3.15, *p* < 0.01] and foreign-accented speech [*t*_(38)_ = 4.51, *p* < 0.001]. The analysis also revealed that while there were no differences in the N400 mean amplitude in the standard condition for either accent over the three regions of analysis, the mean amplitude in the semantic violation condition was different for native and foreign-accented speech in the frontal [*t*_(38)_ = 4.69, *p* < 0.001], central [*t*_(38)_ = 4.89, *p* < 0.001] and posterior region [*t*_(38)_ = 4.53, *p* < 0.001]. Thus, correct sentences in the second block were processed similarly regardless of the accent (regarding the N400 time window, consistently with results of the previous N400 amplitude analysis), and sentences containing semantic violations were processed differently in native and foreign-accented speech.

In sum, this analysis revealed that the N400 effect for semantic violations was significant all over the scalp distribution during foreign-accented speech comprehension. However, this effect was only significant over the posterior region during native speech comprehension. In addition, although the N400 mean amplitude for correct sentences was similar across accents, semantic violations elicited more negative N400 mean amplitudes in foreign-accented speech compared to native speech comprehension all over the scalp. These results suggest that semantic violations were harder to process during foreign-accented speech as compared to native speech comprehension.

### P600: re-analysis processes

We carried out a repeated measures ANOVAs for the P600 component (600–900 ms). This ANOVA included the critical words of the standard and semantic violation conditions of the sentences in the second experimental block. The motivation for this analysis was to check whether foreign-accented speech affected meaning re-analysis processing taking place when listening to semantic violations.

The repeated measures ANOVA for the critical words of the second block included the factors Topography (frontal, central, posterior), Accent (native, foreign), and Semantic status (standard, semantic violation). In this analysis we obtained a significant effect of Topography [*F*_(2, 38)_ = 18.39; *p* < 0.001], and significant interactions between Topography and Accent [*F*_(2, 38)_ = 5.38; *p* < 0.05], and between Accent and Semantic status [*F*_(1, 19)_ = 12.78; *p* < 0.01]. Importantly, the *post-hoc* analysis of the interaction between Accent and Semantic status revealed that the mean amplitude of the P600 component was more positive for semantic violations than for the standard condition only during native speech [native speech, *t*_(19)_ = −3.73, *p* < 0.01; foreign-accented speech, *t*_(19)_ = 0.92, *p* = ns]. This *post-hoc* analysis also revealed that the mean amplitude of the P600 component was more positive during native speech than during foreign-accented speech comprehension for sentences containing a semantic violation [*t*_(19)_ = 2.77, *p* < 0.05]. No differences were observed for the standard condition [*t*_(19)_ = −1.78, *p* = 0.36].

*Post-hoc* analysis of the interaction between Topography and Accent revealed that in the frontal and central regions, the mean amplitude of the P600 component was similar when comprehending native and foreign-accented speech, while in the posterior region the mean amplitude was significantly more positive for native speech as compared to foreign-accented speech [*t*_(38)_ = 2.85, *p* < 0.05]. The *post-hoc* analysis of the interaction between Topography and Accent also showed that the mean amplitude of the P600 components in native speech comprehension was less positive over the frontal region than over central [*t*_(38)_ = −4.54, *p* < 0.001] and posterior regions [*t*_(38)_ = −4.73, *p* < 0.001], with differences also between central and posterior regions [*t*_(38)_ = −4.96, *p* < 0.001]. The same was observed for foreign-accented speech, with significant differences between frontal and central regions [*t*_(38)_ = −2.86, *p* < 0.05], frontal and posterior regions [*t*_(38)_ = −4.85, *p* < 0.001], and central and posterior regions [*t*_(38)_ = −5.30, *p* < 0.001].

**Figure 4:**
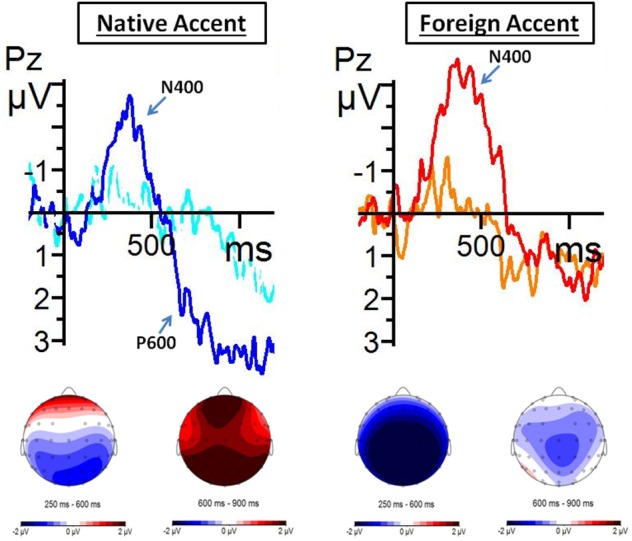
**Grand average ERPs from critical words of Block 2 from Pz electrode**. Averages were extracted for native speech during both standard (blue line) and semantic violation (dark blue line) conditions; and for foreign-accented speech also during standard (orange line) and semantic violation (red line) conditions. Grand average images were extracted at 200 ms before (baseline) and lasting until 1200 ms after the onset of the word. Below, topographic distribution of voltage differences between conditions between 250–600 ms and 600–900 ms after the onset of the critical words.

In sum, this analysis revealed that a widely distributed positivity appeared after the N400 effect for semantic violations in the critical words, although this only occurred during native speech comprehension, not during foreign-accented speech comprehension (Figure 4).

## Discussion

This study aimed at exploring two questions. First, what are the specific mechanisms that native speakers put into play to deal with foreign-accented speech? Previous literature has showed that listeners get better at comprehending foreign-accented speech after a very brief exposure to the accented speakers (Clarke and Garrett, 2004). We examined this issue by looking at the modulation of the P200 and N400 ERP components across two experimental blocks, to clarify whether this improvement takes place at phonetic/acoustic or lexical levels of processing, respectively. Secondly, we explored whether after these changes have taken place, further linguistic processes, such as semantic integration and meaning re-analysis, are affected by foreign-accented speech. This second issue was explored by analyzing the N400 and P600 effects during semantic violation processing in the second experimental block.

In brief, our results show that:

- Foreign-accented speech elicited a less positive P200 as compared to native speech throughout the two experimental blocks. In addition, the P200 mean amplitude was not modulated across the experimental session neither for foreign-accented nor for native speech comprehension.
- The N400 mean amplitude was modulated across exposure to the foreign-accented speech, becoming less negative in the second block of the experiment. This modulation was absent in native speech. Hence, the differences between the N400 mean amplitude between native and foreign-accented speech dissipated after the first experimental block.
- The N400 effect elicited by semantic violations was larger and distributed more widely over the scalp for foreign-accented speech compared to native speech. Furthermore, semantic violations during native speech elicited a widely distributed late positive effect (a P600 effect and a frontal positivity), an effect that was absent during foreign-accented speech comprehension.

We will discuss the implications of these results in more detail below.

### Phonetic/acoustic processing (P200)

In the introduction we argued that an improvement at the extraction of the phonetic/acoustic properties of foreign-accented speech should be indexed by a modulation of the P200 component. This hypothesis was based on previous observations (Reinke et al., 2003; Snyder et al., 2006; De Diego Balaguer et al., 2007; Paulmann et al., 2011), showing that this ERP component is related to the extraction of spectral information and other important acoustic features. Our results are congruent with this view, since the P200 was more negative for foreign-accented speech than for native speech, suggesting that the extraction of spectral information and other acoustic features (such as the information regarding *f*_0_ mean and range) from the former was more difficult (as is also the case with degraded speech; Strauß et al., 2013).

More importantly for our present purposes is the fact that the amplitude of the P200 remained stable across experimental blocks for foreign-accented speech. That is, the extraction of such phonetic/acoustic information remained equally difficult across the experimental session.

A possible limitation of this result is that the P200 component was only analyzed in the first word of the sentences, because this component usually wanes later on at the onset of words embedded in spoken utterances (cf., Kutas and Federmeier (2001), Figure 1; Strauß et al. (2013) also used a similar strategy as our in a study on lexical expectancies under degraded speech). This way, the P200 at sentence onset might index difficulties at identifying the speaker as a foreign speaker, but later on in the sentence the phonetic processing of foreign-accented speech might have improved across experimental blocks. However, since the N400 component already decreased across experimental blocks for the first word of the sentences in foreign-accented speech comprehension, this alternative explanation does not seem applicable.

Taking all this information into account, our results suggest that, at least in the current experimental conditions (and to the extent that the P200 amplitude indexes the extraction of phonetic/acoustic information), rapid improvements do not occur in the extraction of phonetic/acoustic information during foreign-accented speech comprehension. The next question is therefore whether lexical processes actually reveal some sort of adaptation than can help speech comprehension.

### Lexically-driven perceptual learning of foreign-accented speech

As we mentioned in the introduction, most of the studies on perceptual learning propose that this processing is driven by lexical information, which helps listeners to categorize and retune ambiguous phonemes (Norris et al., 2003; Davis et al., 2005; McQueen et al., 2006; Sjerps and McQueen, 2010; Reinisch and Holt, 2014). This way, during lexical processing, listeners would process ambiguous phonemes as representative forms of the original phonemes. We explored this issue by analyzing the modulation of the N400 ERP component across the experiment.

We observed a modulation of the N400 component across the two experimental blocks for foreign-accented speech, which suggests an improvement in lexical-semantic processing. In particular, the fact that the N400 mean amplitude for foreign-accented speech decreases in the second experimental block as compared to the first one could be interpreted as revealing that listeners learned to use lexical information to achieve a better comprehension of foreign-accented speech. Crucially, the N400 mean amplitude for foreign-accented speech decreased across the experimental blocks for the first, critical and final words of the sentences. The fact that there were no differences in the magnitude of the N400 in the second experimental block between the native and the foreign-accented speech conditions is congruent with this interpretation. This interpretation is also consistent with recent studies indicating that listeners are able to use lexical and semantic information during foreign-accented speech comprehension in order to aid online word comprehension, as well as to guide the retuning of their phonetic categories (Trude et al., 2013; Reinisch and Holt, 2014).

Nevertheless, there could be alternative explanations for our observations. One possibility is that attention might have had an effect on the modulations of the N400 ERP component. The differences in the N400 between native and foreign-accented speech comprehension for standard sentences during the first experimental block might be due to more attention being deployed for the foreign-accented speakers. However, under such explanation, we should conclude that attention is devoted to the same extent to native and foreign-accented speech during the second experimental block, since no differences between accent conditions are found in the N400. However, if one takes this view, it remains to be explained why the N400 effect for semantic violations during the second experimental block is larger for foreign-accented than for native speech. Hence, although we cannot exclude differences in attentional processes driving some of our observations, we do not think that such explanation captures the whole set of results.

Importantly, we did not manipulate specific phonetic shifts in the foreign accents. Previous studies on perceptual learning (e.g., Norris et al., 2003) normally used a concrete ambiguous phoneme to which listeners had to adapt. Instead, we used a more general, “ecologically valid” accent scenario, thus suggesting that adaptation to a broader accented speech also occurs due to a lexically-driven top-down mechanism. It is interesting to note that Witteman et al. (2014) observed adaptation to foreign-accented speech even when foreign-accented speakers were inconsistent in their pronunciations (meaning that sometimes foreign-accented speakers produced utterances in a native fashion, whereas other times they produced utterances in a foreign fashion). This would suggest that even in a broad, “ecologically valid,” and more natural scenario (like is our case), in which speakers do not have to produce utterances in the same way multiple times, adaptation is also possible, and it is still ruled by lexical processing. Moreover, since perceptual learning generalizes to words that have not been presented during the training phase (Davis et al., 2005; McQueen et al., 2006; Sjerps and McQueen, 2010), the improvement in processing foreign-accented speech observed in our study was possible even if listeners were presented with new words in new sentences during each trial.

In addition, the absence of any difference between accents in the magnitude of the N400 in the second experimental block suggests that the particular features of our recordings (longer durations for critical words and sentences during foreign-accented speech as compared to native) did not affect the processing of the correct sentences. Furthermore, the fact that the N400 mean amplitude for correct sentences during native speech comprehension did not differ across experimental blocks suggests that semantic violations did not have any effect (such as a surprise effect) on the processing of correct sentences.

Thus, our results, along with other results from previous literature (e.g., Norris et al., 2003; Davis et al., 2005; McQueen et al., 2006; Sjerps and McQueen, 2010; Janse and Adank, 2012; Trude et al., 2013; Banks et al., submitted; Reinisch and Holt, 2014), suggest that lexical information may aid listeners to identify certain pattern variations in the speech of accented speakers. Information at the lexical level would allow listeners to relate their knowledge about the sounds of their native phonological system to sounds that depart from their phonetic/acoustic repertoire (such as the phonetic/acoustic variations in the speech of accented speakers). Furthermore, this lexical information would allow listeners to map these variations onto lexical items, making it possible to the listeners to improve at recognizing, retrieving and integrating the incoming words after brief exposure to the foreign-accented speakers. The improvement in processing foreign-accented speech is reached quickly, and it remains stable in order to be applied to new words in new utterances^6^.

Nevertheless, our data provide no direct behavioral evidence supporting the idea that listeners get better at comprehending foreign-accented speech. Thus, although our results are compatible with previous literature on perceptual learning (Norris et al., 2003; Davis et al., 2005; Reinisch and Holt, 2014), further research combining behavioral results and EEG data would be very enlightening to the field.

### Semantic integration and meaning re-analysis after adaptation to foreign-accented speech

The second issue that we investigated in this study was whether complex linguistic processes, such as semantic integration and meaning re-analysis, were affected in some way by foreign-accented speech after exposure to the accented speech. As we explained before, we took the modulations of the N400 and P600 ERP components elicited by semantic violations during native and foreign-accented speech comprehension as indices of these cognitive processes.

We observed instructive differences between the comprehension of foreign-accented and native speech when the sentences carried a semantic violation. In fact, these violations elicited a larger N400 effect in the context of the comprehension of foreign-accented speech compared to native speech. In addition, the N400 effect for semantic violations during foreign-accented speech comprehension was distributed all over the scalp, while for native speech it only appeared in the classical centro-posterior distribution. This might be due to the lexically-driven perceptual learning of foreign-accented speech. More concretely, a higher demand on lexical processing, needed for the identification and retuning of ambiguous phonetic/acoustic features, might have rendered the effort of accessing the implausible word (and also integrating it in the previous context) extremely difficult.

A potential limitation of our results is the fact that the *f*_0_ mean for semantic violations was higher than for standard words. Nevertheless, since this difference was present both during native and foreign-accented speech, *f*_0_ mean differences between semantic violations and standard words do not seem to account for our pattern of results.

Moreover, the results regarding the modulations of the N400 component during the processing of semantic violations contrast, to some extent, with previous observations by Hanulíková et al. (2012) and Goslin et al. (2012). These two studies have explored the modulations of the N400 component associated with foreign-accented speech in two different contexts. First, Hanulíková et al. (2012) observed similar N400 effects associated with semantic violations irrespective of the accent of the speaker. This is in clear contradiction with our results, in which we found a larger N400 effect for semantic violations during foreign-accented speech comprehension. The difference between these two studies might be explained by the fact that Hanulíková et al. (2012) only used one foreign-accented speaker, with a mild (and highly familiar) accent, possibly making lexical-semantic processing easier. However, it is remarkable that as in Hanulíková et al. (2012), we found a significant N400 effect over the anterior region of the scalp only for foreign-accented speech. That is, while semantic violations during foreign-accented speech comprehension elicited a widely distributed N400 effect, during native speech comprehension semantic violations “only” elicited the classical centro-posterior N400 effect (see e.g., Federmeier and Laszlo, 2009). This might mean that processing semantic violations during foreign-accented speech comprehension requires more cognitive resources than during native speech comprehension.

On the other hand, Goslin et al. (2012) found a less negative N400 component for the final word of sentences produced with a foreign accent as compared to both native and regionally-accented speech. Note that these sentences did not involve semantic violations. Goslin et al. (2012) concluded that listeners tried to anticipate upcoming words in order to avoid difficulties in comprehension. It is important to note the difference that Goslin et al. (2012) reported: their foreign-accented speakers were significantly less intelligible than the native speakers, which is not the case in our, nor Hanulíková et al.'s (2012) study. This way, different levels of intelligibility may lead to different strategies for comprehension, and, therefore, to different modulations at the N400 component, an index of lexical-semantic processing. Future research is needed to distinguish Goslin et al. (2012) and Hanulíková et al. (2012) competing hypotheses.

Regarding the P600 component, which can be related to a second stage of meaning analysis (Kuperberg et al., 2011; Brouwer et al., 2012), we observed a modulation of this ERP component only in the native speech condition. The presence of a P600 modulation during native speech comprehension replicates and extends previous studies in sentence reading (Kuperberg, 2007; Van de Meerendonk et al., 2010). Hence, to the extent that this modulation indexes some sort of meaning re-analysis (Regel et al., 2011; Sanford et al., 2011; Van Petten and Luka, 2012; Martin et al., 2013), our observations would suggest that such re-analysis is not carried out in the foreign-accented speech condition. Importantly, in Hanulíková et al.'s (2012) Figure 3, a large positive deflection over the posterior region of the scalp can be observed during native speech comprehension, following the N400 component elicited by the semantic violation. During foreign-accented speech, this positivity is not present. These results go in the same direction as ours.

A tentative explanation for this absence of the stage of meaning re-analysis is that listeners avoid trying to find an alternative meaning for a semantic violation when it is produced by a foreign-accented speaker. This would be because listeners may treat the semantic violation as an error right away, due to the potential lack of knowledge or fluency of the non-native speaker, hence blocking any re-analysis for alternative meanings. An alternative explanation would be that listeners would lack processing resources during foreign-accented speech comprehension (because of a higher demand on lexical processing) in order to carry out the meaning re-analysis online.

Nevertheless, it is important to remark that the classical P600 component usually has a distribution centered over the posterior areas of the scalp. In our case, the positive effect following the N400 effect was widely distributed. Thornhill and Van Petten (2012) observed that an anterior positivity was elicited by those words that were not highly predictable, independently of the semantic relationship with the expected word. This could mean that during native speech comprehension, semantic violations also elicited a frontal positivity. Thus, listeners would be able to have clear expectations about the upcoming words in an utterance when listening to a native speaker. However, during foreign-accented speech, expectations would not reach the same level of detail. These questions remain for future research on the topic.

## Conclusions

The results of the present study suggest that listeners do not improve at extracting phonetic/acoustic features of foreign-accented speech after brief exposure to it. However, despite this lack of improvement at the extraction of acoustic features, native listeners seem to adapt to the foreign-accented speech due to perceptual learning driven by lexical information. More concretely, lexical information allows listeners to recognize and retune phonetic and acoustic variations onto lexical items, making it possible for the listeners to improve at recognizing, retrieving and integrating the incoming words after brief exposure to the foreign-accented speech. In addition, semantic violations uttered by foreign-accented speakers are harder to process, as compared to semantic violations during native speech comprehension. This is probably because of a higher demand on lexical processing in the retrieval of the non-expected words. Finally, native speech comprehension elicited some sort of meaning re-analysis when semantic violations were present. Such re-analysis seemed to be absent when processing foreign-accented speech, at least under the present experimental conditions.

### Conflict of interest statement

The authors declare that the research was conducted in the absence of any commercial or financial relationships that could be construed as a potential conflict of interest.

## Footnotes

^1^In terms of classic models of lexical access (e.g., Lahiri and Marslen-Wilson, 1991; Pallier et al., 2001), comprehensibility refers to bottom-up activation processes from phonetic representations up to the lexicon to retrieve a possible lexical candidate. In contrast, intelligibility refers to top-down decision processes involving lexical and pragmatic knowledge, arising from the computation of these lexical candidates.

^2^When focusing on the processing of semantic violation in Hanulíková et al.'s (2012) study, a positive deflection is noticeable in the ERP signal after the N400 effect for semantic violations, over the posterior electrodes, only during native speech (cf. Figure 3). This suggests that listeners might also be more tolerant of semantic mistakes when these are produced by foreign-accented speakers. However, Hanulíková et al. (2012) did not report statistical analyses on this effect.

^3^In addition, as a secondary objective, we wanted to explore accent-independent adaptation to foreign-accented speech (taking into account accent strength ratings, not a specific mispronunciation uttered by a specific speaker), along the same lines as Baese-Berk et al. (2013). However, we did not obtain reliable results for accent-independent adaptation (for further details, see Footnote 5).

^4^“Derwing and Munro (1997) reported no relations between intelligibility and comprehensibility for accented speech, using a range of accented varieties of English (Cantonese, Japanese, Polish, and Spanish). Similarly, Weil (2003) failed to find a correlation between reaction-time based measures of comprehensibility collected in a repetition task using Mandarin and Russian accented words, and measures of intelligibility of the same words obtained in a discrimination task. Therefore, an accented speech sample can be rated as highly intelligible, but difficult to process at the same time,” Floccia et al. (2009 p. 380).

^5^We also conducted an analysis taking accent strength ratings into account. In order to do so, we organized these variations in a by-subject parametrical order (from the least to the most foreign accented). The purpose of this analysis was to check to what extent adaptation and semantic processing are sensitive to subtle variations in the intensity of the foreignness of the speakers' accents. However, due to the limitations of the current design and the constraints typical of EEG experiments (small amount of epochs per condition considering sub-division), we did not obtain any reliable result. This issue should be explored in future work with experimental designs specifically focusing on this important question.

^6^Several brain imaging studies investigating phonetic ability (Golestani et al., 2002; Golestani and Zatorre, 2004; Golestani and Pallier, 2007; Golestani et al., 2007) have observed structural as well as functional neural differences correlating with the individual differences in learning speed and success at comprehending and producing second language phonemes. In order to inform about the individual differences in our study, we provide the individual mean amplitudes (over electrode Pz) for each experimental condition (see Supplementary Material, Table 2).

## Supplementary material

Audio 1 **French speaker**.

Audio 2 **Greek speaker**.

Audio 3 **Italian speaker**.

Audio 4 **Japanese speaker**.

Audio 5 **Native speaker 1**.

Audio 6 **Native speaker 2**.

Audio 7 **Native speaker 3**.

Audio 8 **Native speaker 4**.

## References

[B1] Baese-Berk M. M. Bradlow A. R. Wright B. A. (2013). Accent-independent adaptation to foreign accented speech. J. Acoust. Soc. Am. 133, 174–180. 10.1121/1.4789864 23464125 PMC3579861

[B3] Boersma P. Weenink D. (2001). Praat, a system for doing phonetics by computer. Glot Int. 5, 341–345.

[B4] Bradlow A. R. Bent T. (2003). Listener adaptation to foreign-accented English, in Proceedings of the 15th International Congress of Phonetic Sciences, eds Sole M. J. Recasens D. Romero J. (Barcelona), 2881–2884

[B5] Bradlow A. R. Bent T. (2008). Perceptual adaptation to non-native speech. Cognition 106, 707–729. 10.1016/j.cognition.2007.04.005 17532315 PMC2213510

[B6] Brouwer H. Fitz H. Hoeks J. (2012). Getting real about semantic illusions: rethinking the functional role of the P600 in language comprehension. Brain Res. 1446, 127–143. 10.1016/j.brainres.2012.01.055 22361114

[B7] Burkhardt P. (2006). Inferential bridging relations reveal distinct neural mechanisms: evidence from event-related brain potentials. Brain Lang. 98, 159–168. 10.1016/j.bandl.2006.04.005 16725188

[B8] Burkhardt P. (2007). The P600 reflects cost of new information in discourse memory. Neuroreport 18, 1851–1854. 10.1097/WNR.0b013e3282f1a999 18090325

[B9] Clarke C. M. (2000). Perceptual adjustment to foreign-accented English. J. Acoust. Soc. Am. 107, 2856 10.1121/1.429245

[B10] Clarke C. M. Garrett M. F. (2004). Rapid adaptation to foreign-accented English. J. Acoust. Soc. Am. 116, 3647–3658. 10.1121/1.1815131 15658715

[B11] Cuetos F. Glez-Nosti M. Barbón A. Brysbaert M. (2011). SUBTLEX-ESP: Spanish word frequencies based on film subtitles. Psicológica 32, 132–143 Available online at: http://crr.ugent.be/papers/CUETOS%20et%20al%202011.pdf

[B12] Davis M. H. Johnsrude I. S. Hervais-Adelman A. Taylor K. McGettigan C. (2005). Lexical information drives perceptual learning of distorted speech: evidence from the comprehension of noise-vocoded sentences. J. Exp. Psychol. 134, 222–241. 10.1037/0096-3445.134.2.222 15869347

[B13] De Diego Balaguer R. Toro J. M. Rodriguez-Fornells A. Bachoud-Lévi A. C. (2007). Different neurophysiolocal mechanisms underlying word and rule extraction from speech. PLoS ONE 2:e1175. 10.1371/journal.pone.0001175 18000546 PMC2063512

[B14] DeLong K. A. Groppe D. M. Urbach T. P. Kutas M. (2012). Thinking ahead or not? Natural aging and anticipation during reading. Brain Lang. 121, 226–239. 10.1016/j.bandl.2012.02.006 22406351 PMC3571658

[B15] DeLong K. A. Urbach T. P. Kutas M. (2005). Probabilistic word preactivation during language comprehension inferred from electrical brain activity. Nat. Neurosci. 8, 1117–1121. 10.1038/nn1504 16007080

[B16] Derwing T. M. Munro M. J. (1997). Accent, intelligibility and comprehensibility. Stud. Second Lang. Adq. 19, 1–16 10.1017/S0272263197001010

[B17] Federmeier K. D. Laszlo S. (2009). Time for meaning: electrophysiology provides insights into the dynamics of representation and processing in semantic memory, in The Psychology of Learning and Motivation, ed Ross B. H. (San Diego, CA: Elsevier), 1–44.

[B18] Floccia C. Butler J. Goslin J. Ellis L. (2009). Regional and foreign accent processing in English: can listeners adapt? J. Psycholinguist. Res. 38, 379–412. 10.1007/s10936-008-9097-8 19117134

[B19] Fraser H. (2000). Coordinating improvements in pronunciation teaching for adult learners of English as a second Language, in DETYA (ANTA Innovative Project), (Canberra). 1–53.

[B20] Golestani N. Molko N. Dehaene S. LeBihan D. Pallier C. (2007). Brain structure predicts the learning of foreign speech sounds. Cereb. Cortex 17, 575–582. 10.1093/cercor/bhk001 16603709

[B21] Golestani N. Pallier C. (2007). Anatomical correlates of foreign speech sound production. Cereb. Cortex 17, 929–934. 10.1093/cercor/bhl003 16740583

[B22] Golestani N. Paus T. Zatorre R. J. (2002). Anatomical correlates of learning novel speech sounds. Neuron 35, 494–506. 10.1016/S0896-6273(02)00862-0 12372292

[B23] Golestani N. Zatorre R. J. (2004). Learning new sounds of speech: recollocation of neural substrates. Neuroimage 21, 494–506. 10.1016/j.neuroimage.2003.09.071 14980552

[B24] Goslin J. Duffy H. Floccia C. (2012). An ERP investigation of regional and foreign accent processing. Brain Lang. 122, 92–102. 10.1016/j.bandl.2012.04.017 22694999

[B25] Gullick M. M. Priya M. Coch D. (2013). Imagining the truth and the moon: an electrophysiological study of abstract and concrete word processing. Psychophysiology 50, 431–440. 10.1111/psyp.12033 23445520

[B26] Gut U. (2012). The LeaP corpus: a multilingual corpus of spoken learner German and learner English, in Multilingual corpora and multilingual corpus analyses, eds Schmidt T. Wömer K. (Amsterdam: John Benjamins), 3–23.

[B27] Hanulíková A. van Alphen P. M. van Goch M. M. Weber A. (2012). When one person's mistake is another's standard usage: the effect of foreign accent on syntactic processing. J. Cogn. Neurosci. 24, 878–887. 10.1162/jocn_a_00103 21812565

[B28] Hanulíková A. Weber A. (2012). Sink positive: linguistic experience with th substitutions influences nonnative word recognition. Atten. Percept. Psychophys. 74, 613–629. 10.3758/s13414-011-0259-7 22207311

[B29] Hay J. Nolan A. Drager K. (2006). From fush to feesh: exemplar priming in speech perception. Linguist. Rev. 23, 351–379 10.1515/TLR.2006.014

[B30] Holcomb P. J. Grainger J. O'Rourke T. (2002). An electrophysiological study of the effects of orthographic neighborhood size on printed word perception. J. Cogn. Neurosci. 14, 938–950. 10.1162/089892902760191153 12191460

[B31] Janse E. Adank P. (2012). Predicting foreign-accent adaptation in older adults. Q. J. Exp. Psychol. 65, 1563–1585. 10.1080/17470218.2012.658822 22530648

[B32] Jilka M. (2000). The Contribution of Intonation to the Perception of Foreign Accent. PhD. dissertation, Arbeiten des Institus für Maschinelle Sprachverabeitung, Universität Stuttgart.

[B33] Kroll J. F. Merves J. S. (1986). Lexical access for concrete and abstract words. J. Exp. Psychol. 12, 92–107 10.1037/0278-7393.12.1.92

[B34] Kuperberg G. R. (2007). Neural mechanisms of language comprehension: challenges to syntax. Brain Res. Spec. Issue 1146, 23–49. 10.1016/j.brainres.2006.12.063 17400197

[B35] Kuperberg G. R. Paczynski M. Ditman T. (2011). Establishing causal coherence across sentences: an ERP study. J. Cogn. Neurosci. 23, 1230–1246. 10.1162/jocn.2010.21452 20175676 PMC3141815

[B36] Kutas M. Federmeier K. D. (2001). Electrophysiology reveals semantic memory use in language comprehension. Trends Cogn. Sci. 4, 463–470. 10.1016/S1364-6613(00)01560-6 11115760

[B37] Kutas M. Hillyard S. A. (1984). Brain potentials during reading reflect word expectancy and semantic association. Nature 307, 161–163. 10.1038/307161a0 6690995

[B38] Kutas M. Lindamood T. E. Hillyard S. A. (1984). Word expectancy and event-related brain potentials during sentence processing, in Preparatory States and Processes, eds Kornblum S. Requin J. (Hillsdale NJ: Erlbaum), 217–237.

[B39] Lahiri A. Marslen-Wilson W. (1991). The mental representation of lexical form: a phonological approach to the recognition of lexicon. Cognition 38, 245–294. 10.1016/0010-0277(91)90008-R 2060271

[B40] Lane H. (1963). Foreign accent and speech distortion. J. Acoust. Soc. Am. 35, 451–453 10.1121/1.1918501

[B41] Laszlo S. Federmeier K. D. (2011). The N400 as a snapshot of interactive processing: evidence from regression analyses of orthographic neighbor and lexical associate effects. Psychophysiology 48, 176–186 10.1111/j.1469-8986.2010.01058.x PMC2955840 20624252

[B42] Lau E. F. Phillips C. Poeppel D. (2008). A cortical network for semantics: (de)constructing the N400. Nat. Rev. Neurosci. 9, 920–933. 10.1038/nrn2532 19020511

[B43] Martin C. D. Thierry G. Kuipers J. R. Boutonnet B. Foucart A. Costa A. (2013). Bilinguals reading in their second language do not predict upcoming words as native readers do. J. Mem. Lang. 69, 574–588 10.1016/j.jml.2013.08.001

[B44] McNamara T. P. (2005). Semantic Priming: Perspectives from Memory and Word Recognition. New York, NY: Psychology Press.

[B45] McQueen J. M. Cutler A. Norris D. (2006). Phonological abstraction in the mental lexicon. Cogn. Sci. 30, 1113–1126. 10.1207/s15516709cog0000_79 21702849

[B46] Munro M. J. Derwing T. M. (1995a). Foreign accent, comprehensibility, and intelligibility in the speech of second language learners. J. Acoust. Soc. Am. 45, 73–97.

[B47] Munro M. J. Derwing T. M. (1995b). Processing time, accent, and comprehensibility in the perception of native and foreign-accented speech. Lang. Speech 38, 289–306. 8816082 10.1177/002383099503800305

[B48] Neely J. H. (1991). Semantic priming effects in visual word recognition: a selective review of current findings and theories, in Basic Processes in Reading. Visual Word Recognition, eds Besner D. Humphreys G. W. (Hillsdale, NJ: Lawrence Erlbaum Associates, Inc.), 264–336.

[B49] Nissen S. L. Dromey C. Wheeler C. (2007). First and second language tongue movements in Spanish and Korean bilingual speakers. Phonetica 64, 201–216. 10.1159/000121373 18421243

[B50] Norris D. McQueen J. M. Cutler A. (2003). Perceptual learning in speech. Cogn. Psychol. 47, 204–238. 10.1016/S0010-0285(03)00006-9 12948518

[B51] Pallier C. Colomé A. Sebastián-Gallés N. (2001). The influence of native-language phonology on lexical access: concrete exemplar-based vs. abstract lexical entries. Psychol. Sci. 12, 445–449. 10.1111/1467-9280.00383 11760129

[B52] Paulmann S. Ott D. V. M. Kotz S. A. (2011). Emotional speech perception unfolding in time: the role of the basal ganglia. PLoS ONE 6:e17694. 10.1371/journal.pone.0017694 21437277 PMC3060083

[B53] Regel S. Gunter T. C. Friederici A. D. (2011). Isn't it ironic? An electrophysiological exploration of figurative language processing. J. Cogn. Neurosci. 23, 277–293. 10.1162/jocn.2010.21411 20044894

[B54] Reinisch E. Holt L. L. (2014). Lexically-guided phonetic retuning of foreign-accented speech and its generalization. J. Exp. Psychol. Hum. Percept. Perform. 40, 539–555. 10.1037/a0034409 24059846 PMC3962813

[B55] Reinisch E. Weber A. (2012). Adapting to supresegmental lexical stress errors in foreign-accented speech. J. Acoust. Soc. Am. 132, 1165–1172. 10.1121/1.4730884 22894235

[B56] Reinke K. S. He Y. Wang C. Alain C. (2003). Perceptual learning modulates sensory evoked response during vowel segregation. Cogn. Brain Res. 17, 781–791. 10.1016/S0926-6410(03)00202-7 14561463

[B57] Rossi S. Hartmüller T. Vignotto M. Obrig H. (2013). Electrophysiological evidence for modulation of lexical processing after repetitive exposure to foreign phonotactic rules. Brain and Lang. 127, 404–414. 10.1016/j.bandl.2013.02.009 23489581

[B58] Rothermich K. Schmidt-Kassow M. Kotz S. A. (2012). Rhythm's gonna get you: regular meter facilitates semantic sentence processing. Neuropsychologia 50, 232–244. 10.1016/j.neuropsychologia.2011.10.025 22178743

[B59] Sanford A. J. Leuthold H. Bohan J. Sanford A. J. S. (2011). Anomalies at the borderline of awareness: an ERP study. J. Cogn. Neurosci. 23, 514–523. 10.1162/jocn.2009.21370 19925201

[B60] Schmid P. M. Yeni-Komshian G. H. (1999). The effects of speaker accent and target predictability on perception of mispronunciations. J. Speech Lang. Hear. Res. 42, 56–64. 10.1044/jslhr.4201.56 10025543

[B61] Sidaras S. K. Alexander J. E. D. Nygaard L. C. (2009). Perceptual learning of systematic variation in Spanish-accented speech. J. Acoust. Soc. Am. 125, 3306–3316. 10.1121/1.3101452 19425672 PMC2736743

[B62] Sjerps M. J. McQueen J. M. (2010). The bounds of flexibility in speech perception. J. Exp. Psychol. Hum. Percept. Perform. 36, 195–211. 10.1037/a0016803 20121304

[B63] Smith M. E. Halgren E. (1987). Event-related potentials during lexical decision: effects of repetition, word frequency, pronounceability, and concreteness. Electroencephalogr. Clin. Neurophysiol. Suppl. 40, 417–421. 3480158

[B81] Snyder J. S. Alain C. Picton T. W. (2006). Effects of attention on neuroelectric correlates of auditory stream segregation. J. Cogn. Neurosci. 18, 1–13. 10.1162/089892906775250021 16417678

[B64] Strauß A. Kotz S. A. Obleser J. (2013). Narrowed expectancies under degraded speech: revisiting the N400. J. Cogn. Neurosci. 25, 1383–1395. 10.1162/jocn_a_00389 23489145

[B65] Thornhill D. E. Van Petten C. (2012). Lexical versus conceptual anticipation during sentence processing: frontal positivity and N400 ERP components. Int. J. Psychophysiol. 83, 382–392. 10.1016/j.ijpsycho.2011.12.007 22226800

[B66] Trude A. M. Tremblay A. Brown-Schmidt S. (2013). Limitations on adaptation to foreign accents. J. Mem. Lang. 69, 349–367. 10.1016/j.jml.2013.05.002 24014935 PMC3763963

[B67] Van de Meerendonk N. Kolk H. H. J. Vissers C. T. W. M. Chwilla D. J. (2010). Monitoring in language perception: mild and strong conflicts elicit different ERP patterns. J. Cogn. Neurosci. 22, 67–82. 10.1162/jocn.2008.21170 19199401

[B68] Van Petten C. Kutas M. (1991). Electrophysiological evidence for the flexibility of lexical processing, in Word and Sentence, ed Simpson G. (Amsterdam: North Holland Press), 129–174.

[B69] Van Petten C. Luka B. J. (2006). Neural localization of semantic context effects in electromagnetic and hemodynamic studies. Brain Lang. 97, 279–293. 10.1016/j.bandl.2005.11.003 16343606

[B70] Van Petten C. Luka B. J. (2012). Prediction during language comprehension: benefits, costs, and ERP components. Int. J. Psychophysiol. 83, 176–190. 10.1016/j.ijpsycho.2011.09.015 22019481

[B80] van Wijngaarden S. J. (2001). Intelligibility of native and non-native Dutch speech. Speech Commun. 35, 103–113 10.1016/S0167-6393(00)00098-4

[B71] Wade T. Jongman A. Sereno J. (2007). Effects of acoustic variability in the perceptual learning of non-native-accented speech sounds. Phonetica 64, 122–144. 10.1159/000107913 17914280

[B72] Weber A. Di Betta A. M. McQueen J. M. (2014). Treack or trit: adaptation to genuine and arbitrary foreign accents by monolingual and bilingual listeners. J. Phon. 46, 34–51 10.1016/j.wocn.2014.05.002

[B73] Weil S. A. (2001). Foreign accented speech: encoding and generalization. J. Acoust. Soc. Am. 109, 2473 10.1121/1.4744779

[B74] Weil S. A. (2003). The Impact of Perceptual Dissimilarity on the Perception of Foreign Accented Speech. Unpublished dissertation, The Ohio State University.

[B75] West W. C. Holcomb P. J. (2000). Imaginal, semantic, and surface level processing of concrete and abstract words: an electrophysiological investigation. J. Cogn. Neurosci. 12, 1024–1037. 10.1162/08989290051137558 11177422

[B76] Wester F. Gilbers D. Lowie W. (2007). Substitution of dental fricatives in English by Dutch L2 speakers. Lang. Sci. 29, 477–491 10.1016/j.langsci.2006.12.029

[B77] Witteman M. J. Weber A. McQueen J. M. (2013). Foreign accent strength and listener familiarity with an accent codetermine speed of perceptual adaptation. Atten. Percept. Psychophys. 75, 537–556. 10.3758/s13414-012-0404-y 23456266

[B78] Witteman M. J. Weber A. McQueen J. M. (2014). Tolerance for inconsistency in foreign-accented speech. Psychon. Bull. Rev. 21, 512–519. 10.3758/s13423-013-0519-8 24234167

[B79] Wlotko E. W. Federmeier K. D. (2013). Two sides of meaning: the scalp-recorded N400 reflects distinct contributions from the cerebral hemispheres. Front. Psychol. 4:181. 10.3389/fpsyg.2013.00181 23630506 PMC3632783

