# Supplementary material for: Processing changes when listening to foreign-accented speech
Source: Front Hum Neurosci. 2015 Mar 25;9:167. doi: 10.3389/fnhum.2015.00167 (PMC4373278; doi:10.3389/fnhum.2015.00167)
Supplement: Supplementary file 9 [file Image5.PDF]

Figure 5. Spectrographic representations of the Audio samples.

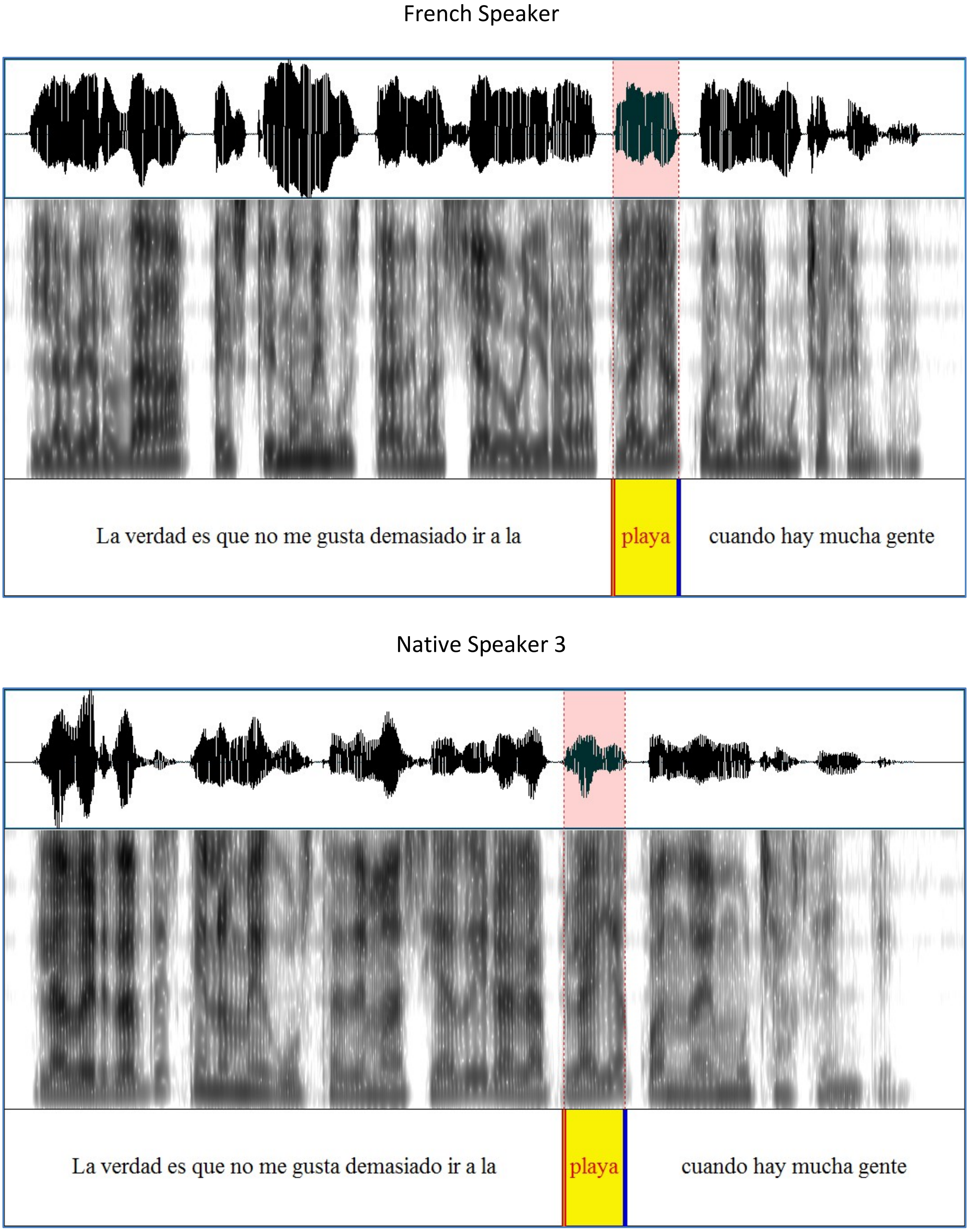

"The truth is that I do not like coming to the ***beach*** *when it is very crowded."*

Greek Speaker

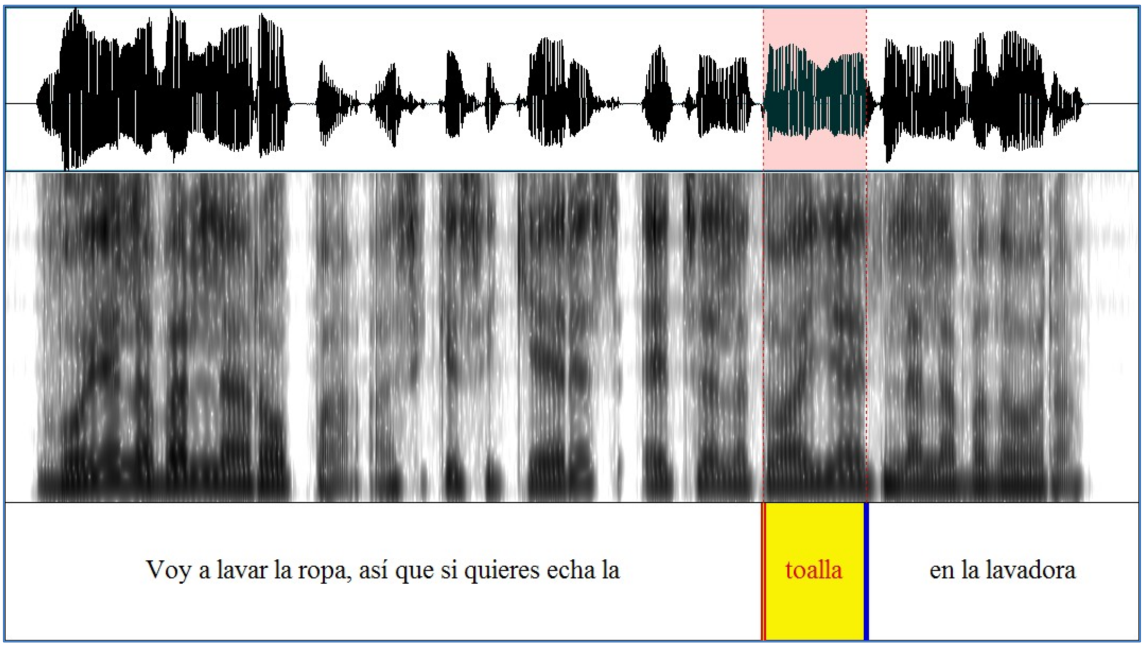

Native Speaker 2

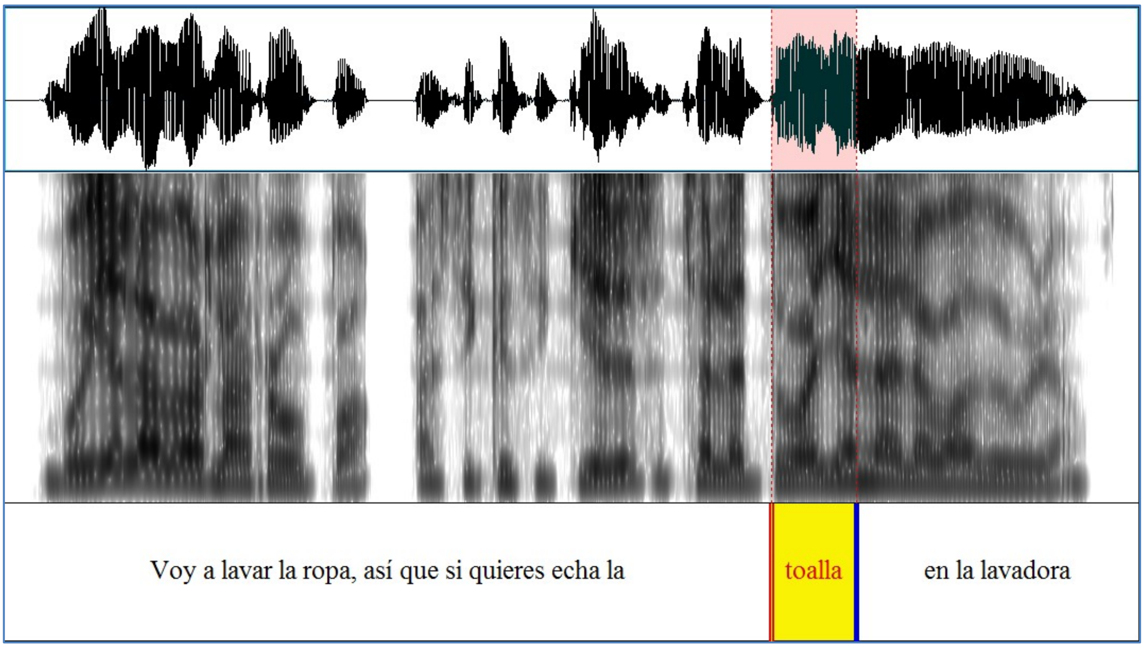

"I'm doing the laundry, so put the ***towel*** *in the washing machine if you want."*

Italian Speaker

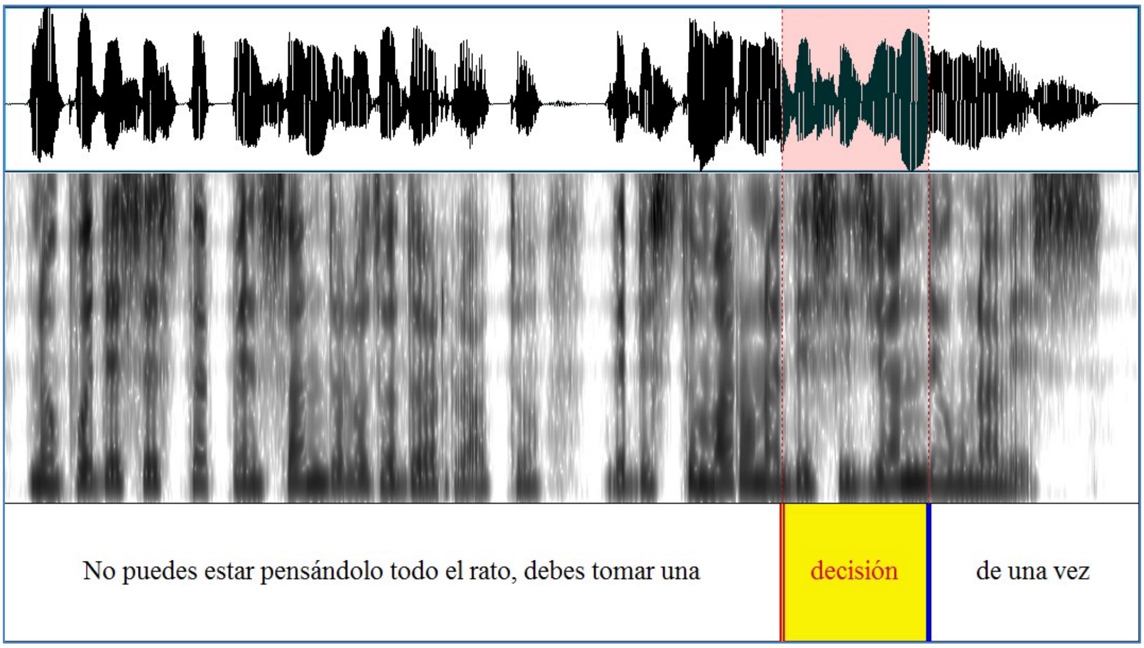

Native Speaker 1

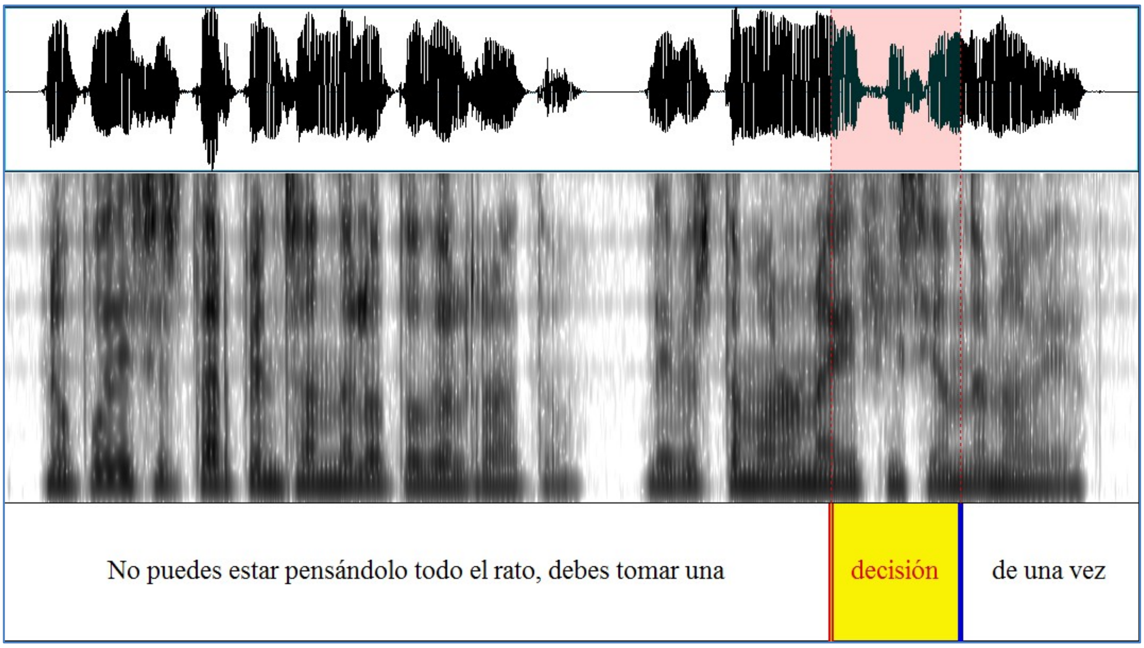

"You cannot be thinking all the time, you must make a ***decision*** *eventually."*

Japanese Speaker

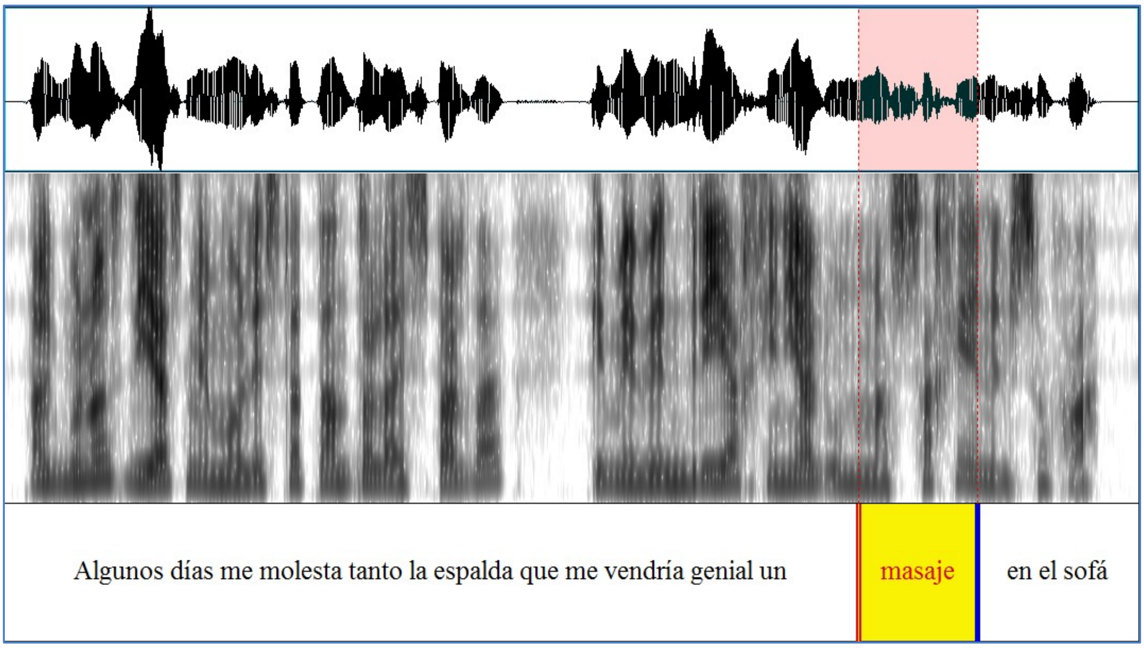

Native Speaker 4

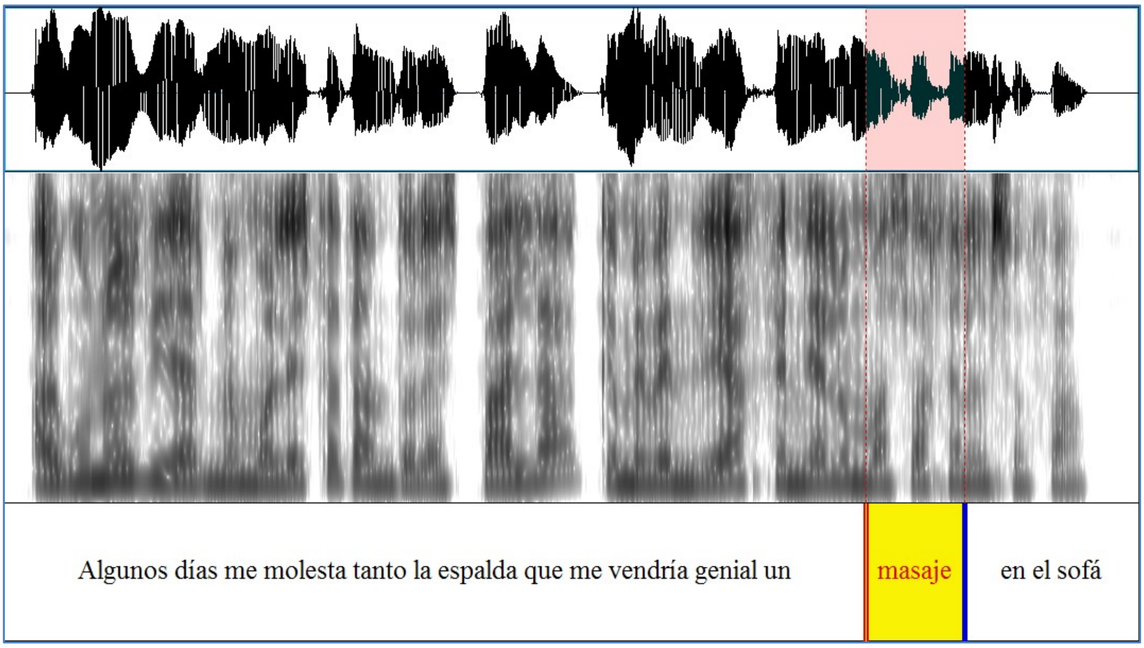

"Some days my back hurts so much that I could use a ***massage*** *in the couch."*
