# Supplementary material for: Processing changes when listening to foreign-accented speech
Source: Front Hum Neurosci. 2015 Mar 25;9:167. doi: 10.3389/fnhum.2015.00167 (PMC4373278; doi:10.3389/fnhum.2015.00167)
Supplement: Supplementary file 10 [file Table2.PDF]

	P200				N400														P600			
	First Word				*First Word*				*Critical Word*						*Final Word*				*Critical Word*			
	Block1		*Block 2*		*Block1*		*Block 2*		*Block1 Standard*		*Block 2 Standard*		*Block 2 SV*		*Block1*		*Block 2*		*Block2 Standard*		*Block2 SV*	
	Native	Foreign	Native	Foreign	Native	Foreign	Native	Foreign	Native	Foreign	Native	Foreign	Native	Foreign	Native	Foreign	Native	Foreign	Native	Foreign	Native	Foreign
S01	-3,12	-6,63	-4,75	-6,60	-9,07	**-11,50**	-7,50	**-7,98**	-1,21	**-0,84**	-1,16	**0,03**	-2,18	-5,61	1,93	3,47	2,54	3,17	-3,45	0,26	-0,01	-3,49
S02	-3,21	-4,16	-3,13	-3,17	-6,15	**-7,54**	-6,11	**-4,26**	-0,40	1,68	0,95	0,74	-2,50	-0,33	6,57	**2,62**	4,02	**2,92**	2,80	3,80	3,71	3,25
S03	0,76	1,51	-1,34	-1,42	-2,26	-1,88	-3,98	-4,30	2,96	1,44	1,12	0,19	-2,53	-3,89	-0,27	**-1,62**	0,54	**2,73**	-3,82	1,37	1,03	-0,25
S04	2,93	3,17	-0,34	1,17	2,38	**1,37**	-1,36	**0,69**	-0,12	**-2,36**	-1,23	**-1,75**	-3,46	-4,56	-0,18	**0,04**	2,56	**0,32**	-0,25	-1,23	1,03	-2,79
S05	-1,56	-1,99	-1,82	-2,91	-6,03	-4,99	-4,10	-5,47	4,52	**0,21**	0,09	**4,95**	-4,14	-2,81	6,15	**1,04**	1,75	**1,62**	2,90	3,30	3,23	3,60
S06	-2,72	0,35	0,72	-1,26	-0,53	**-2,74**	-1,27	**-1,75**	-1,14	**2,30**	-1,69	**2,81**	0,64	-3,77	2,29	**-0,80**	3,61	**-0,45**	-1,64	1,60	7,30	-1,02
S07	3,75	2,68	0,00	-0,28	-1,46	-2,53	-2,26	-4,74	0,78	2,38	1,28	0,14	1,34	-0,63	-0,62	**-1,62**	-0,68	**0,91**	1,80	2,80	5,70	2,14
S08	-2,15	-3,02	0,05	-1,82	-6,56	**-5,93**	-3,15	**-5,40**	1,71	**-0,14**	0,15	**0,95**	-0,87	-3,14	1,27	6,25	3,44	5,69	1,71	2,33	4,75	2,40
S09	-1,06	-1,41	2,22	2,54	-1,34	**-1,60**	0,07	**1,02**	-1,63	**-1,23**	-1,77	**-0,64**	-3,56	-2,91	-1,82	**-2,05**	-0,49	**1,13**	-1,47	-1,01	-1,00	-1,69
S10	0,25	0,49	-1,89	-0,53	-1,67	**-4,87**	-3,49	**-3,42**	-0,32	**-0,98**	0,18	**-0,86**	3,15	-1,26	-1,69	**-0,52**	2,83	**0,21**	2,18	2,06	5,09	4,02
S11	1,50	3,76	3,01	2,26	-1,12	**0,05**	0,15	**1,43**	-1,35	-1,30	0,70	-1,65	-1,11	-2,02	2,22	0,32	0,31	-0,82	1,24	-1,30	2,55	2,76
S12	-1,16	-1,14	1,52	1,52	-2,66	**-2,62**	0,29	**0,10**	-2,15	0,79	0,55	-0,52	-2,42	-2,58	0,29	0,16	1,43	-0,59	2,11	1,67	3,29	0,92
S13	-2,03	-4,68	-1,02	-2,68	-5,30	**-5,37**	-3,51	**-3,70**	-2,58	**-1,82**	2,68	**1,49**	-4,90	-4,07	1,84	0,86	-0,02	-1,35	3,12	2,24	-0,87	-1,68
S14	1,45	-0,38	-0,05	2,19	1,63	**-0,81**	-1,63	**0,87**	2,75	0,01	-0,94	-2,77	-0,11	-1,49	-0,27	-1,03	2,15	-2,22	-0,99	-1,17	2,95	-1,57
S15	-1,57	-4,99	-1,55	-2,81	-3,25	**-1,87**	-0,91	**-0,44**	0,34	**-2,17**	-0,93	**-1,36**	-1,27	0,25	2,94	1,27	2,00	1,12	-1,06	-1,14	0,04	3,86
S16	4,47	3,09	0,20	-0,20	0,59	-1,63	-3,12	-2,82	1,17	**1,63**	-0,02	**2,23**	-0,19	0,67	1,75	0,83	-1,61	-0,29	-0,20	0,83	5,89	1,69
S17	-0,08	-0,99	-0,54	-1,55	-0,82	**-3,83**	-1,08	**-2,42**	-4,08	-0,07	-2,42	-1,58	0,14	-1,59	1,89	**-1,15**	2,72	**0,11**	-1,54	0,70	2,21	1,55
S18	-1,23	-1,96	-3,18	-2,18	-4,33	**-4,92**	-3,95	**-3,06**	-1,03	**-2,14**	-1,59	**-0,17**	-1,12	-1,25	0,90	**1,50**	1,86	**1,61**	-1,52	0,68	0,32	0,19
S19	-2,61	-4,71	-2,06	-1,33	-3,90	**-6,18**	-4,34	**-1,56**	1,33	**1,15**	2,30	**1,83**	-4,85	-1,26	5,52	6,22	4,31	4,53	3,25	2,30	1,08	2,22
S20	5,08	5,83	4,17	1,35	-1,38	-2,16	-2,50	-3,32	2,20	-4,32	2,12	-7,01	4,48	-4,92	3,18	**0,56**	0,61	**3,86**	-2,63	-2,98	-1,41	-5,86
**Mean**	-0,12	-0,76	-0,49	-0,89	-2,66	-3,58	-2,69	-2,53	0,09	-0,29	0,02	-0,15	-1,27	-2,36	1,69	0,82	1,69	1,21	0,13	0,85	2,34	0,51
**SE**	0,57	0,76	0,48	0,51	0,65	0,66	0,46	0,57	0,47	0,40	0,33	0,54	0,56	0,39	0,53	0,52	0,37	0,46	0,51	0,41	0,57	0,61

Table 2

Mean amplitude values over electrode Pz for each subject and each one of the experimental conditions. Standard = standard semantic condition; SV = semantic violation semantic condition; SE = standard error. Values underlined are those in which the N400 was lower in Block 2, as compared to Block 1, during foreign-accented speech comprehension (standard semantic condition). Note that during First Word comprehension, 75% of the participants showed this pattern over electrode Pz (an N400 mean amplitude more positive during Block 2 as compared to Block 1). The amount of participants showing this pattern over electrode Pz decreased in posterior sentence regions (Critical Word = 60% of the participants; Final Word = 55% of the participants), probably because of further semantic and wrap-up processes interacting with the possibility of an improvement at processing foreign-accented speech. In any case, all participants showed the decreasing N400 pattern for foreign-accented speech comprehension over electrode Pz at one word position at least, and 65% of the participants showed this pattern at two word positions at least.
